# An opposing function of paralogs in balancing developmental synapse maturation

**DOI:** 10.1371/journal.pbio.2006838

**Published:** 2018-12-26

**Authors:** Plinio D. Favaro, Xiaojie Huang, Leon Hosang, Sophia Stodieck, Lei Cui, Yu-zhang Liu, Karl-Alexander Engelhardt, Frank Schmitz, Yan Dong, Siegrid Löwel, Oliver M. Schlüter

**Affiliations:** 1 European Neuroscience Institute Göttingen, University Medical Center, Göttingen, Germany; 2 Collaborative Research Center 889, University of Göttingen, Göttingen, Germany; 3 Department of Neuroscience, University of Pittsburgh, Pittsburgh, Pennsylvania, United States of America; 4 Department of Systems Neuroscience, Universität Göttingen, Göttingen, Germany; 5 Department of Psychiatry and Psychotherapy, University Medical Center, Göttingen, Germany; 6 Department of Neuroanatomy, Medical School, Saarland University, Homburg, Germany; Duke University, United States of America

## Abstract

The disc-large (DLG)–membrane-associated guanylate kinase (MAGUK) family of proteins forms a central signaling hub of the glutamate receptor complex. Among this family, some proteins regulate developmental maturation of glutamatergic synapses, a process vulnerable to aberrations, which may lead to neurodevelopmental disorders. As is typical for paralogs, the DLG-MAGUK proteins postsynaptic density (PSD)-95 and PSD-93 share similar functional domains and were previously thought to regulate glutamatergic synapses similarly. Here, we show that they play opposing roles in glutamatergic synapse maturation. Specifically, PSD-95 promoted, whereas PSD-93 inhibited maturation of immature α-amino-3-hydroxy-5-methyl-4-isoxazole propionic acid–type glutamate receptor (AMPAR)–silent synapses in mouse cortex during development. Furthermore, through experience-dependent regulation of its protein levels, PSD-93 directly inhibited PSD-95’s promoting effect on silent synapse maturation in the visual cortex. The concerted function of these two paralogs governed the critical period of juvenile ocular dominance plasticity (jODP), and fine-tuned visual perception during development. In contrast to the silent synapse–based mechanism of adjusting visual perception, visual acuity improved by different mechanisms. Thus, by controlling the pace of silent synapse maturation, the opposing but properly balanced actions of PSD-93 and PSD-95 are essential for fine-tuning cortical networks for receptive field integration during developmental critical periods, and imply aberrations in either direction of this process as potential causes for neurodevelopmental disorders.

## Author summary

During development, there are restricted time windows in which the brain’s cortex is reorganized; these changes depend on experience and serve to establish functionality. In immature brains, silent synapses—newly born synapses that are inactivated due to a lack of normal receptor-mediated signaling—are frequent. Recent studies have suggested that silent synapses help to reorganize the connection pattern between excitatory neurons. However, it remains largely unknown how functions are established during critical periods and what specific cellular processes contribute to these changes. Here, we analyze silent synapses in mice and show that two very similar proteins, postsynaptic density (PSD)-93 and PSD-95, which evolved via duplication of a single ancestral gene, have opposing roles in the conversion of synapses from an immature state to an active state. While PSD-95 promotes maturation, PSD-93 acts as a brake and slows the process. Consequently, in mice lacking PSD-93, the fraction of silent synapses declines faster, and a critical period of visual cortex development closes too early. Visual acuity is not affected in mice that lack either PSD-93 or PSD-95, but is severely reduced if mice are deficient of both proteins. In contrast, proper orientation discrimination in adult mice required both PSD-93 and PSD-95. These results indicate that these two proteins have related and balancing functions that are crucial for normal development of synapses and optimal brain function.

## Introduction

The postsynaptic density (PSD) is a proteinaceous network that regulates and coordinates the signaling of multiple receptors and other proteins in a confined region at the synapse, including developmental changes, to reach its mature functionality. Most proteins of the glutamate receptor complex are evolutionarily diversified as paralogous proteins [1]. A common notion is that this diversification enables specific adaptations of protein functions in increasingly complex organisms [2,3]. In particular, paralogs either adapted for specific requirements of different cellular compartments or organs, such as the liver- or heart-specific lactate dehydrogenases, or evolved more specialized functions in the same compartment, such as the opsins for color vision in photoreceptor cells [4,5]. However, it remains elusive whether the multiple paralogous proteins of the PSD functionally interact within the same synapse or each individually predominates in different synapses.

The importance of individual PSD paralogs in synapse maturation is highlighted by the observation that genetic variants in single genes cause neurodevelopmental disorders, including autism spectrum disorders (ASDs) [6] and schizophrenia [7]. Focusing on PSD-95, a core protein of the glutamate receptor signaling complex, we recently demonstrated that PSD-95–dependent maturation of α-amino-3-hydroxy-5-methyl-4-isoxazole propionic acid (AMPA)-type glutamate receptor (AMPAR)-silent synapses ends the critical period of the juvenile form of ocular dominance (OD) plasticity (ODP) in the visual cortex [8], a classical model for experience-dependent critical period plasticity [9–11]. The experience-dependent silent synapse maturation may thus serve as a model mechanism for studying the specific role of PSD-95 and its paralogs of glutamate receptor complexes in synapse and circuit maturation during developmental critical periods. Furthermore, sensory defects are typical for neurodevelopmental disorders, including schizophrenia and autism [12–14]. Thus, given the similar cytoarchitecture of functional domains of the neocortex, mechanistic insights into sensory cortical phenotypes likely also translate to pathomechanisms of mental disorders.

As a paralog of PSD-95, PSD-93 also directly interacts with glutamate receptors and controls AMPAR synaptic trafficking [15–17]. The gene *Dlg2*, coding for PSD-93, contains six different N-terminal isoforms [18,19]. Allelic variants and somatic mosaicism of *Dlg2* are associated with schizophrenia and other neurodevelopmental disorders [20–22]. The analysis of PSD-93 loss and gain of function in different brain regions has uncovered partly conflicting results that so far prevented a clear understanding of the physiological function of the paralogs PSD-93 and PSD-95 in the regulation of glutamatergic synapses, and what might go awry in neurodevelopmental disorders [19,23–25].

Here, we show that PSD-93 and PSD-95 regulate experience-dependent maturation of silent synapses in an opposing manner in that PSD-95 promotes, while PSD-93 inhibits, the maturation. Concurrently, critical period closure is impaired in PSD-95 knock-out (KO) mice [8], while it closes precociously in PSD-93 KO mice. The lack of either paralog impaired the functional optimization of cortical networks and resulted in the impairment of visual perception with visual acuity remaining intact, indicating a dissociation of developmental processes for perception and vision. In PSD-93/95 double KO (dKO) mice—lacking both of the promoting and inhibiting effects—silent synapse maturation proceeded in terms of maturation speed more similar to but mechanistically distinct from wild-type (WT) mice. Consequently, visual features were compromised with impaired visual acuity. Thus, PSD-95 and PSD-93 functionally cooperate in constructive silent synapse maturation with opposing roles in the glutamate receptor complex, with either paralog promoting vision, while requiring their opposing function for fine-tuning perceptual capabilities. The opposing function of paralogs extends the repertoire of evolutionary functional specialization and provides a conceptual framework for the analysis of paralog-specific pathomechanisms in neurodevelopmental disorders.

## Results

### Opposing roles of PSD-93 and PSD-95 in silent synapse maturation in the visual cortex

**Fig 1:**
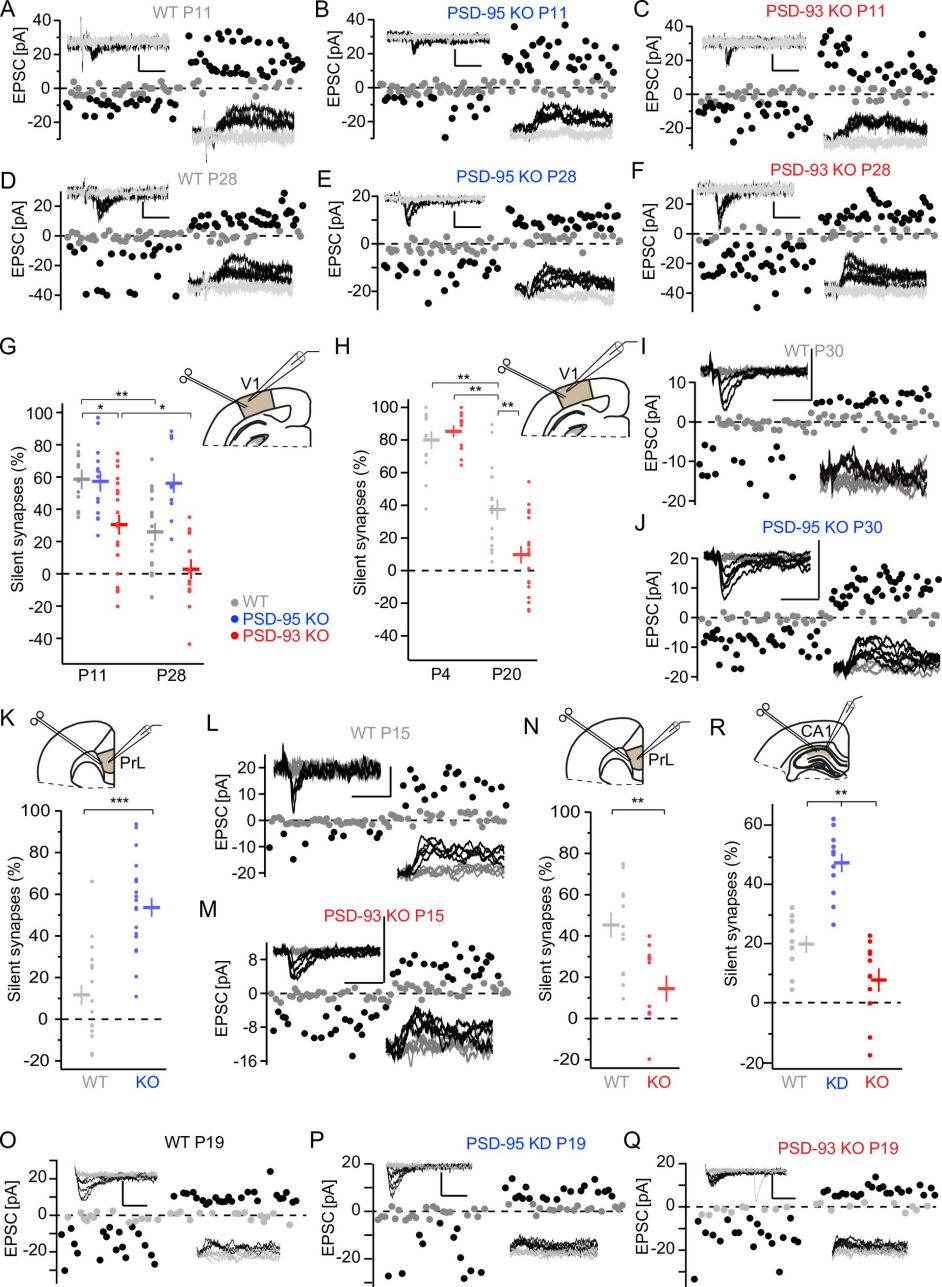
Accelerated developmental decrease of AMPA-silent synapses in the cortex of PSD-93 KO mice. (A–F, I, J, L, M, O–Q) Sample traces of pyramidal neuron EPSCs from different brain areas using minimal stimulation of afferents of mice with different genotypes and ages. Sample traces (insets) and analysis of the peak values of AMPA receptor EPSCs, recorded at V_h_ = −60 mV (downward deflection), or composite glutamate receptor EPSCs, recorded at V_h_ = +40 mV (upward deflection), of successes (black) and failures (gray) of individual EPSCs are depicted. Scale bar: 20 ms and 25 pA. (A-F) Sample traces from V1 layer 2/3 pyramidal neurons with L4 afferents of P11/P28 WT (A/D), PSD-95 KO (B/E), and PSD-93 KO (C/F) mice. (G-H) Summary graphs of the fraction of silent synapses of WT (gray), PSD-95 KO (blue), and PSD-93 (red) mice at different developmental time points. Dots represent values of single neurons; horizontal bars are genotype averages. Recording scheme of layer 2/3 pyramidal neuron of V1 in coronal brain slices is depicted in inset, with patch pipette and bipolar stimulating electrode. *p < 0.05, **p < 0.01. See also S1 Fig. Underlying data for this figure can be found in S1 Data. (I, J, L, M) Sample traces of mPFC layer 2/3 pyramidal neuron with white matter afferents of P30/P15 WT (I/L), P30 PSD-95 KO (J), and P15 PSD-93 KO (M). (K, N) Summary graphs of the fraction of silent synapses of WT (gray), PSD-95 KO (blue), and PSD-93 KO (red) mice at different developmental time points. Recording scheme of layer 2/3 pyramidal neuron of the mPFC PrL in coronal brain slices is depicted in inset, with patch pipette and bipolar stimulating electrode. **p < 0.01, ***p < 0.001. Underlying data for this figure can be found in S1 Data. (O-Q) Sample traces of hippocampal CA1 pyramidal neuron with Schaffer collateral afferents of P20 WT (O), PSD-95 KD (P), and PSD-93 KO (Q). (R) Summary graphs of the fraction of silent synapses of WT (gray), PSD-95 KD (blue), and PSD-93 KO (red) mice at P20. Recording scheme of CA1 pyramidal neuron of V1 in coronal brain slices is depicted in inset, with patch pipette and bipolar stimulating electrode. **p < 0.01. Underlying data for this figure can be found in S1 Data. AMPA, α-amino-3-hydroxy-5-methyl-4-isoxazole propionic acid; CA1, Cornu Ammonis 1; EPSC, excitatory postsynaptic current; KD, knock-down; KO, knock-out; L4, layer 4; mPFC, medial prefrontal cortex; P, postnatal day; PrL, prelimbic cortex; PSD, postsynaptic density; V_h_, holding potential; V1, primary visual cortex; WT, wild-type.

Loss of function of PSD-95 impairs silent synapse maturation in the visual cortex [8,26]. Reassuring this previous finding, we observed in PSD-95 KO mice that the fraction of silent synapses in the layer 4 (L4)–to–layer 2/3 pyramidal neurons of mouse visual cortex before eye opening at postnatal day (P) 11 was similar to that in WT mice (*F*_5,92_ = 12.06; *p* < 0.001; P11: WT versus PSD-95 KO, *p* = 1.0; Fig 1A, 1B and 1G). While in WT mice, the fraction of silent synapses decreased after eye opening, the fraction did not change in PSD-95 KO mice (WT: P11 versus P28, *p* < 0.01; PSD-95 KO: P11 versus P28, *p* = 1.0; Fig 1D–1E and 1G). In contrast, in PSD-93 KO, the fraction of silent synapses at P11 was smaller compared with that of WT and PSD-95 KO mice (P11: WT versus PSD-93 KO, *p* < 0.05; PSD-93 KO versus PSD-95 KO, *p* < 0.05; Fig 1C and 1G). In PSD-93 KO mice, the fraction of silent synapses progressively decreased but was approaching 0%, instead of approximately 25%, in WT mice at P28 (PSD-93 KO: P11 versus P28, *p* < 0.05; Fig 1F and 1G). While loss of PSD-95 prevented the developmental decrease in silent synapses, the effect of loss of PSD-93 was opposite by accelerating this developmental decrease. Thus, the developmental trajectories of silent synapses were different in response to loss of function of PSD-93 versus PSD-95 (two-factor ANOVA; genotype: *F*_2,92_ = 17.3, *p* < 0.001; age: *F*_1,92_ = 13.2, *p* < 0.001; interaction: *F*_2,92_ = 3.94, *p* < 0.05).

To further characterize the time course of the accelerated developmental trajectory of silent synapses in PSD-93 KO mice, we assessed the fraction of silent synapses at two additional time points, after birth (P4) and at the beginning of the critical period (P20). At P4, the fraction of silent synapses was high in both WT and PSD-93 KO mice, with similar percentages (*F*_3,60_ = 41.2, *p* < 0.001; P4: WT versus PSD-93 KO, *p* = 0.94; Fig 1H). At P20, the fraction of silent synapses in both genotypes declined, but PSD-93 KO mice exhibited much lower levels of silent synapses compared with WT mice (P20: WT versus PSD-93 KO, *p* < 0.01; Fig 1H). Thus, the developmental trajectory of silent synapses of both genotypes starts at a similar value at birth but declines at an accelerated pace in PSD-93 KO mice, reaching toward 0% already during the critical period (S1 Fig). In contrast, the fraction of silent synapses in PSD-95 KO mice remained constantly high and did not decline from P11 until P28, nor further throughout the critical period into late adulthood [8].

### Opposing roles of PSD-93 and PSD-95 in silent synapse maturation in hippocampus and medial prefrontal cortex

Previous studies reported different effects of loss of PSD-93 on synaptic AMPARs and N-methyl-D-aspartate (NMDA)-type glutamate receptors (NMDARs) in different brain regions [19,23–25]. In the cortex of PSD-93 KO mice, the number of synaptic NMDARs is reduced [25]. In the hippocampus of PSD-93 KO mice, one study reported a reduction of synaptic AMPARs, while others did not observe this effect [19,23,24]. Therefore, we investigated whether loss of PSD-93 generally reduces the fraction of silent synapses in cortical synapses of principal neurons and whether loss of PSD-95 impairs their maturation. We assessed the fraction of silent synapses in layer 2/3 pyramidal neurons of the medial prefrontal cortex (mPFC) and Cornu Ammonis 1 (CA1) pyramidal neurons of the hippocampus. At P30 in the mPFC of PSD-95 KO mice, the fraction of silent synapses was higher compared with that of WT mice (*t* = −5.45; *p* < 0.001; Fig 1K). Conversely, at P15 in the mPFC of PSD-93 KO mice, the fraction of silent synapses was reduced compared with that of WT mice (*t* = 3.35; *p* < 0.01; Fig 1N). Similarly, at P20 in the hippocampus of mice with loss of PSD-95 through a short hairpin RNA (shRNA)-mediated knock-down (KD), or in PSD-93 KO mice, the fraction of silent synapses was increased or decreased compared with that of WT mice, respectively (*F*_2,30_ = 38.1, *p* < 0.01; WT versus PSD-95 KD, *p* < 0.01; WT versus PSD-93 KO, *p* < 0.05; PSD-95 KD versus PSD-93 KO, *p* < 0.01; Fig 1R). Together, these results reveal that in all assessed pyramidal neurons, PSD-95 is necessary for the maturation of AMPAR-silent synapses, while, without PSD-93, their fraction is reduced.

### Experience dependence of silent synapse maturation in the visual cortex

**Fig 2:**
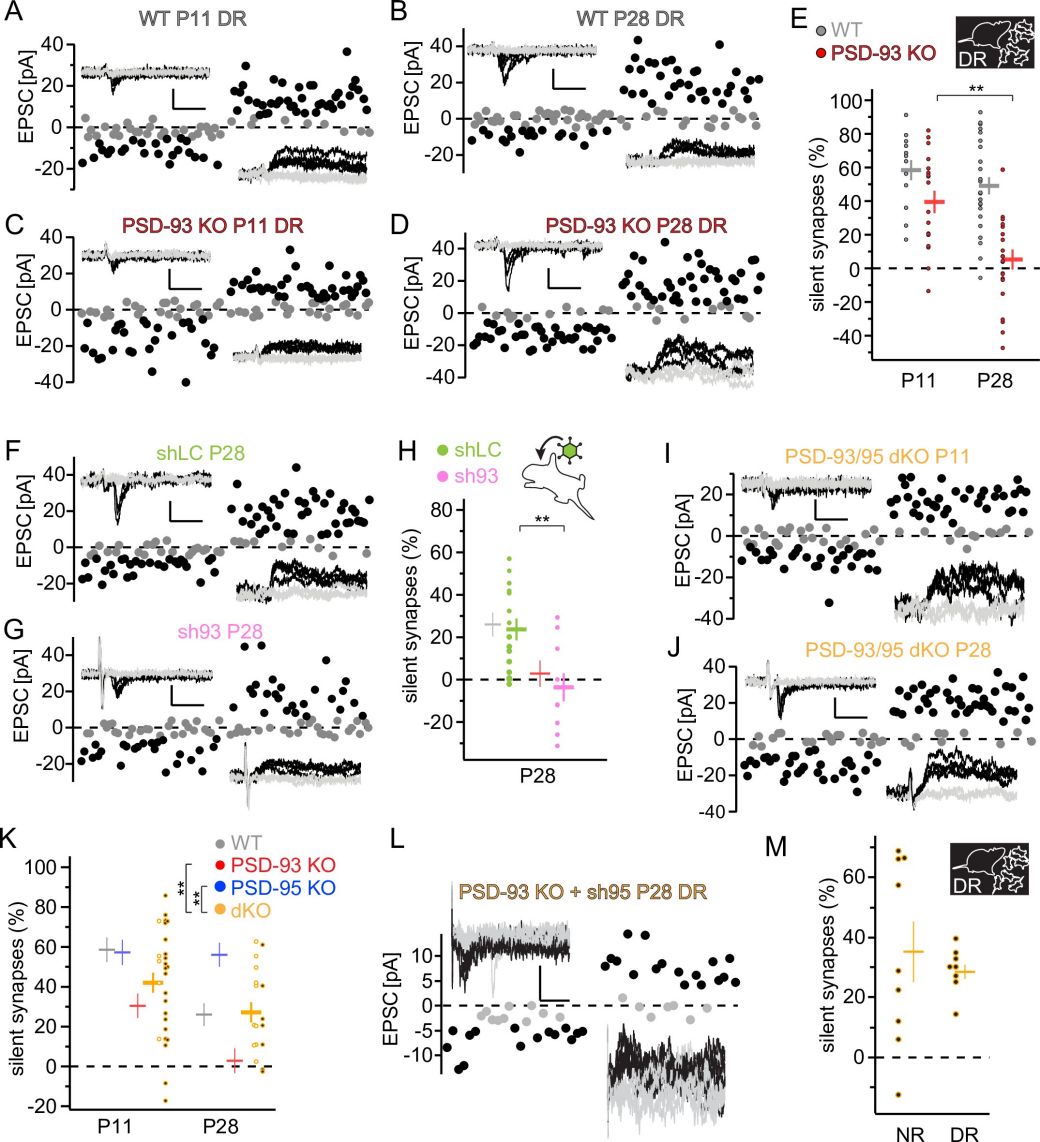
Visual experience and DLG-MAGUK paralogs are required for the developmental decrease of silent synapses. (A-D, F, G, I, J, L) Sample traces of EPSCs using minimal stimulation of L4 afferents of DR WT mice at P11/P28 (A/B), DR PSD-93 KO (93KO) mice at P11/P28 (C/D), P28 WT mice with AAV-shLC/AAV-sh93 (F/G), PSD-93/95 dKO mice at P11/P28 (I/J), or DR at P28. Scale bar: 20 ms and 25 pA. (E, H, K, M) Summary graphs of the fraction of silent synapses of indicated genotypes/manipulation (inset) at indicated developmental time points. Dots represent values of single neurons. (K, M) Values of dKO (orange) and PSD-93 KO with sh95 are presented with white- or black-centered dots, respectively. Values of dKO and PSD-93 with sh95 are pooled for average value (horizontal bar). For comparison, average values from Fig 1 are plotted as thin horizontal bars without dots of individual values (H, K). Statistical difference between individual groups are presented in graphs and between genotypes in inset. Data are displayed as in Fig 1. **p < 0.01. Underlying data for this figure can be found in S1 Data. AAV, adeno-associated viral vector; dKO, double KO; DLG, disc-large; DR, dark-reared; EPSC, excitatory postsynaptic current; KO, knock-out; L4, layer 4; MAGUK, membrane-associated guanylate kinase; NR, normal-reared; P, postnatal day; PSD, postsynaptic density; shLC, short hairpin RNA against luciferase; sh93, short hairpin RNA against PSD-93; sh95, short hairpin RNA against PSD-95; WT, wild-type.

Because in PSD-93 KO mice, the fraction of silent synapses was already reduced compared with WT mice at eye opening (about P12), we tested whether the accelerated developmental decrease resulted from visual experience. In dark-reared (DR) WT mice, the fraction of silent synapses did not decline between P11 and P28 (*F*_3,80_ = 15.7, *p* < 0.001; WT: P11 versus P28, *p* = 0.73, Fig 2A, 2B and 2E), indicating that the decline of silent synapses after eye opening was visual experience dependent [27]. However, lack of experience through dark rearing did not affect the accelerated decline of silent synapses in PSD-93 KO mice (PSD-93 KO: P11 versus P28, *p* < 0.01; Fig 2C–2E), indicating that by removing PSD-93, the developmental decrease of silent synapses became independent of visual experience and remained accelerated.

Notably, at P4, the fraction of silent synapses was about 80% and thus higher than at P11 (about 55%; Fig 1). Both DR and normal-reared (NR) WT mice exhibited similar fractions of silent synapses at P11 (Figs 1 and 2), indicating that before eye opening, the fraction of silent synapses declines independently of visual experience. Furthermore, because in PSD-95 KO mice, the fraction of silent synapses stays at the eye-opening level, mechanisms to decrease the fraction of silent synapses before eye opening are apparently intact, but visual experience–dependent maturation after eye opening is absent, indicating two different mechanisms for the decrease of silent synapses (Fig 1) [8].

We then tested whether the accelerated maturation of silent synapses by loss of PSD-93 was cell autonomous. Using low-titer recombinant adeno-associated viral vectors (AAV) expressing an shRNA against PSD-93 (sh93) or short hairpin luciferase (shLC) as a control [8,19], we sparsely transduced a low fraction of primary visual cortex (V1) neurons. On P28, in AAV-sh93–expressing layer 2/3 pyramidal neurons, the fraction of silent synapses was smaller than that of AAV-shLC–expressing ones (*t* = 3.85, *p* < 0.01; Fig 2F–2H). Similar to PSD-93 KO, the fraction of silent synapses in AAV-sh93–transduced neurons was about 0% at P28, while the values in AAV-shLC–expressing control neurons was about 25%, similar to WT mice (Fig 2H). Taken together, these results reveal that the PSD-93–dependent acceleration of silent synapse maturation is cell autonomous rather than a consequence of compensatory network mechanisms.

### Developmental decrease of silent synapses in the absence of PSD-93 and PSD-95

Our results so far reveal opposing functions of PSD-95 and PSD-93 on the decrease of silent synapses in the developing visual cortex. To further examine this conclusion, we measured the time course of silent synapses in PSD-93/95 dKO mice. The survival rate of newborn dKO mice was low [23]. Thus, to generate sufficient numbers of mutant mice lacking both PSD-93 and -95 in the visual cortex, we analyzed two types of mutant mice in parallel. When available, we used dKO mice from PSD-93 KO and PSD-95 heterozygous breeding and, alternatively, mice with a combination of genetic KO of PSD-93 and AAV-mediated KD of PSD-95. For the latter approach, we injected an AAV expressing shRNA against PSD-95 (sh95) into the visual cortex of P0 PSD-93 KO mice [8]. We validated this approach by comparing the fraction of silent synapses between the two manipulations. At both P11 and P28, the fraction of silent synapses between PSD-93/95 dKO and PSD-93 KO with sh95, respectively, was similar (P10: dKO versus PSD-93 KO/sh95, *t* = 0.577, *p* = 0.58; P28: dKO versus PSD-93 KO/sh95, *t* = 0.378, *p* = 0.71), indicating that a cell selective loss of both proteins and loss in all neurons had a similar effect.

At P4, we used the low-yield PSD-93/95 dKO mice, as P4 did not allow sufficient time for AAV-mediated expression. The fraction of silent synapses was similar to that of WT mice (84.5% ± 2.8%, *n* = 5; S1 Fig). Likewise, at P10 and P28, in PSD-93/95-lacking neurons, the fraction of silent synapses was similar to that of WT mice, but higher than that of PSD-93 KO mice and smaller than that of PSD-95 KO mice (two-way ANOVA: WT versus dKO/KD, *F*_1,77_ = 0.182, *p* = 0.18; PSD-95 KO versus dKO/KD, *F*_1,69_ = 13.3, *p* < 0.001; PSD-93 KO versus dKO/KD, *F*_1,79_ = 9.24, *p* < 0.005; Fig 2I–2K). These results further support our hypothesis that PSD-93 and PSD-95 prevent and promote the developmental decrease of AMPA-silent synapses, respectively. However, in neurons lacking both paralogs, the time course of silent synapse decrease was similar to that of WT mice, indicating that developmental decrease of silent synapses is also achieved without these proteins.

To test whether this decrease in the absence of the two paralogs is mechanistically different than that in their presence, we measured the developmental time course of silent synapses in DR mice. In DR PSD-93 KO/sh95 mice, the fraction of silent synapses at P28 was similar to that of NR PSD-93 KO/sh95 mice (*t* = 0.64; *p* = 0.54; Fig 2L and 2M), indicating that in the absence of both paralogs, the decrease of silent synapses was independent of visual experience. Thus, the mechanisms of silent synapse decrease differ between WT and KOs. While in WT mice, the developmental decrease of silent synapses is experience dependent, it progresses both before eye opening and in the absence of PSD-93 or the absence of both paralogs, independently of experience.

### Precocious closure of critical period plasticity in PSD-93 KO mice

**Fig 3:**
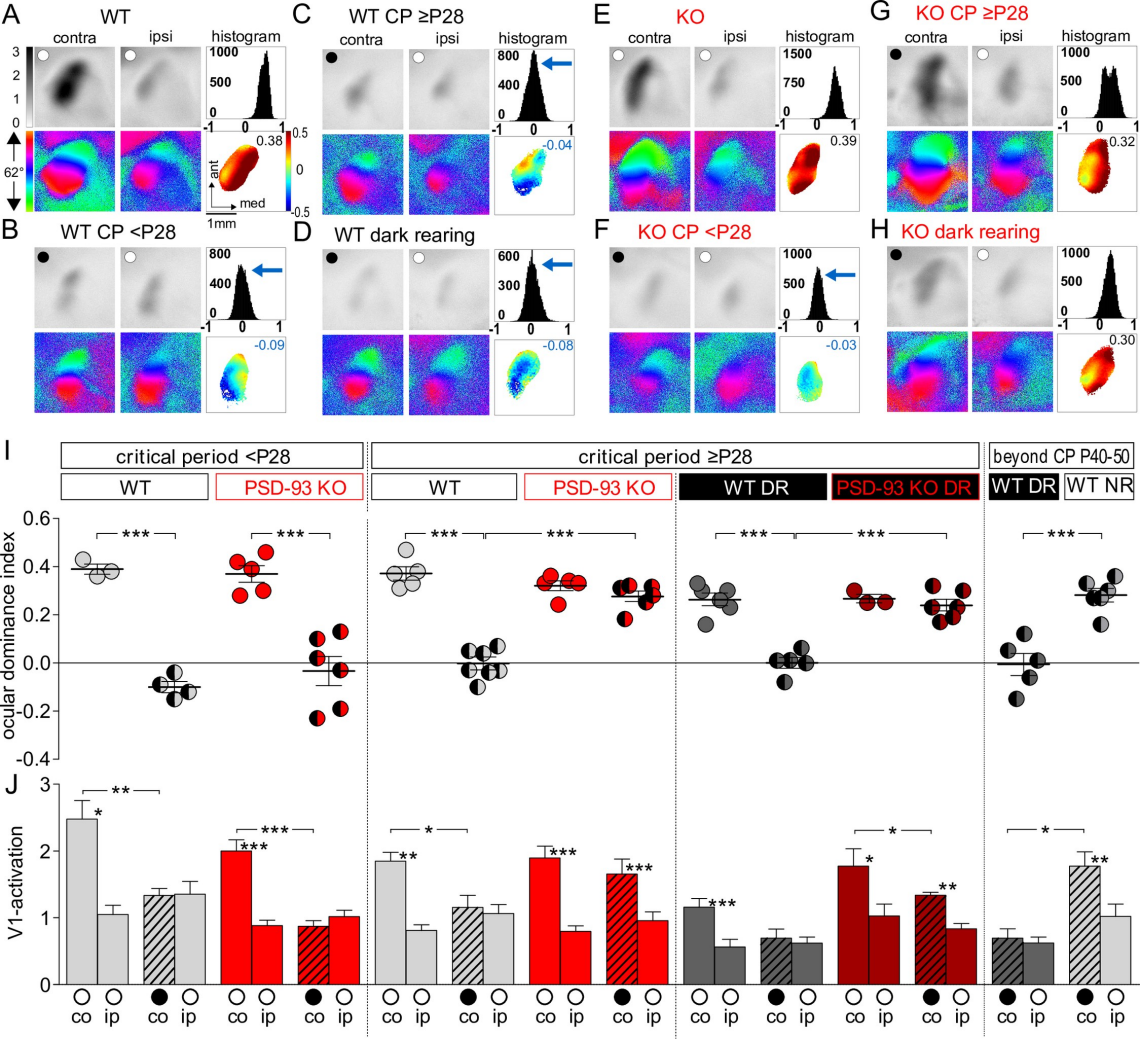
Precocious closure of the critical period for juvenile ODP in PSD-93 KO mice. (A-H) Optically imaged activity and retinotopic maps in V1 of WT (A–D) and PSD-93 KO mice (E-H) during mid CP (<P28 = P24–P27), during late CP (≥P28 = P28–P35), and beyond the CP (P40–P50) before (A and E) and after 4 d of MD (WT: B-D and KO: F-H). V1 images of DR WT and PSD-93 KO mice after 4 d MD are illustrated in D and H. Grayscale coded response magnitude maps (top rows, expressed as fractional change in reflectance ×10^−4^), color-coded maps of retinotopy (bottom rows), histogram of OD scores (top right of panels), and color-coded OD maps (bottom right, including average ODI) are illustrated. In control mice of both genotypes (A and E), activity patches evoked by stimulation of the contralateral (contra) eye were always darker than those evoked by the ipsilateral eye (ipsi) stimulation, the average ODI was positive, and warm colors prevailed in the two-dimensional OD maps, indicating contralateral dominance. After 4 d of MD (MD eye illustrated as black spot), there was an OD shift towards the open eye in WT mice during mid (B) and late CP (C), and beyond the CP after dark rearing (D), whereas PSD-93 KO mice only showed an OD shift during mid CP (F), which was already absent in late CP (G) and not rescued after dark rearing (H). After MD in WT mice, the ODI histogram shifted leftwards (blue arrows), the ODI decreased, and colder colors prevailed in the OD maps (negative ODI values). In contrast, in PSD-93 KO mice, OD plasticity was absent beyond P28 (compare F and G), and the deprived, contra eye continued to dominate V1 (G). (I, J) Summary graph of ODI (I) and V1 activation (J). ○, open eyes; ●, deprived eyes. ODIs (I) and V1 activation (J) before (oo) and after (●o) 4 d MD in PSD-93 KO (red) and WT mice (light gray). Values of DR mice are illustrated in darker colors. Symbols in I represent ODI values of individuals; means are marked by horizontal lines. (J) V1 activation elicited by stimulation of the contra (co) or ipsi (ip) eye. Note that juvenile OD plasticity persisted in WT mice after the CP (beyond CP) after dark rearing (WT DR), but not in NR WT mice, whereas it was absent in both late CP (≥P28) and DR (≥P28) PSD-93 KO mice. Furthermore, all OD shifts after 4 d of MD were primarily mediated by reductions of deprived eye responses in V1 (J). *p < 0.05, **p < 0.01, ***p < 0.001. Underlying data for this figure can be found in S1 Data. ant, anterior; co, contralateral; contra, contralateral; CP, critical period; DR, dark-reared; ip, ipsilateral; ipsi, ipsilateral; KO, knock-out; MD, monocular deprivation; med, medial; NR, normal-reared; ODI, ocular dominance index; ODP, ocular dominance plasticity; P, postnatal day; PSD, postsynaptic density; V1, primary visual cortex; WT, wild-type.

A classical test for experience-dependent cortical plasticity in mammals is ODP in V1, which is induced by closing one eye (monocular deprivation [MD], an experimental model of a cataract) [10]. In mouse V1, neurons in the binocular region of V1 predominantly respond to sensory inputs from the contralateral eye (contra) and to lesser extend to the ipsilateral eye (ipsi). During the critical period, mouse V1 is susceptible to activity-dependent refinement of neural circuits and establishment of critical visual functions, particularly receptive field integration, including binocular vision [10,28]. In standard cage-raised mice, a brief (4-d) MD induces an OD shift of visually evoked responses in V1 towards the open eye [8,29,30]. This juvenile ODP (jODP) is mediated by a reduction of deprived eye responses in the binocular part of V1 and is temporally confined to the critical period [31,32]. We previously reported that PSD-95–dependent silent synapse maturation is required for the closure of the jODP in mice [8]. Using PSD-93 KO mice, we explored the potential reverse correlation that precocious silent synapse maturation leads to a precocious termination of the critical period for OD plasticity. We performed MD for 4 d at two different time points during the critical period, in mid critical period (P24–P27), when silent synapses in PSD-93 KO were still present, and in late critical period (P28–P35), when the fraction of silent synapses was approaching 0% (Fig 2). During both time periods, V1 of PSD-93 KO and WT control mice was dominated by visual inputs from the contralateral eye, and the OD index (ODI) was positive, measured with optical imaging of intrinsic signals (pre-P28, no MD ODI: WT versus KO; *p* = 0.66; post-P28, no MD ODI: WT versus KO, *p* = 0.17; Fig 3A, 3E, 3I and 3J).

In contrast to standard cage-raised WT mice that expressed jODP after 4 d of MD at both time points (Fig 3B, 3C, 3I and 3J), PSD-93 KO mice expressed jODP only before P28, but not later (Fig 3F, 3G, 3I and 3J). Before P28, the shift in the ODI in WT mice was mediated by a reduction of deprived (contra) eye responses in V1 (no MD versus MD, *p* < 0.01; Fig 3B, 3C, 3I and 3J), which is characteristic for jODP during the critical period [31,32], whereas ipsi-evoked responses in V1 did not change (no MD versus MD, *p* = 0.29). The change in ODI in PSD-93 KO mice before P28 was similar to that of WT mice (ODI: WT MD versus KO MD, *p* = 0.42, Fig 3B, 3F, 3I and 3J) and also mediated by a reduction of deprived (contra) eye responses in V1 (KO no MD versus KO MD, *p* < 0.001; Fig 3F, 3I and 3J). Ipsi-evoked responses did not change (KO no MD versus KO MD, *p* = 0.31). These results indicate that jODP plasticity itself does not require PSD-93.

In PSD-93 KO mice after P28 (≥P28), a 4-d MD was unable to induce an OD shift towards the open eye (*p* = 0.17; Fig 3G), whereas age-matched WT mice continued to express OD plasticity (*p* < 0.001), which was also mediated by a reduction of deprived (contra) eye responses in V1, as expected in the critical period (WT no MD versus WT MD, *p* < 0.05). Thus, while jODP is expressed in PSD-93 KO mice, its critical period terminates precociously.

In DR WT mice, the critical period for jODP is prolonged [33]. Because in PSD-93 KO mice, silent synapse maturation was not halted by dark rearing, we tested whether jODP would be. This was not the case. Even in the critical period, DR PSD-93 KO mice did not show jODP (ODI: MD versus no MD, *p* = 0.5; Fig 3H and 3I), whereas DR WT mice exhibited jODP both during the critical period (ODI: MD versus no MD, *p* < 0.001; Fig 3I) and also after the critical period (P40–P50), in contrast to NR WT mice (ODI: DR MD versus NR MD, *p* < 0.001, Fig 3I). The OD shift of DR WT mice exhibited a clear trend for a reduction of deprived (contra) eye responses (no MD ≥P28 versus MD ≥P28, *p* = 0.052; Fig 3D and 3H and 3J), whereas ipsi-evoked responses in V1 remained unchanged (no MD ≥P28 versus MD ≥P28, *p* = 0.74). Together, these results reveal that jODP does not require PSD-93, because before P28, jODP was expressed in PSD-93 KO mice, whereas the lack of jODP after P28 was correlated with the precocious decrease of silent synapses. Furthermore, dark rearing, which halts silent synapse maturation in WT mice and prolongs the critical period of jODP, neither halted the decrease in silent synapses nor prolonged the critical period of jODP in PSD-93 KO mice, revealing a strict correlation between silent synapse decrease and the closure of the critical period for jODP in the visual cortex.

The basic organization of the brain is normal in PSD-93 KO mice [34]. To test whether the visual cortex of PSD-93 KO mice is similarly organized as in WT mice, we analyzed optically recorded V1 activity and retinotopic maps by stimulating the contralateral eye with either horizontally or vertically moving bars (S2A and S2D Fig). V1 activation (elevation: WT versus KO, *p* = 0.993; azimuth: WT versus KO, *p* = 0.22; S2G and S2I Fig) and retinotopic map quality (map scatter) (elevation: WT versus KO, *p* = 0.573; azimuth: WT versus KO, *p* = 0.197; S2H and S2J Fig) were similar in PSD-93 KO and WT mice, indicating that basic visual activation of V1 is not altered in the absence of PSD-93.

### Visual cortex–restricted PSD-93 KD precociously terminates juvenile ODP

**Fig 4:**
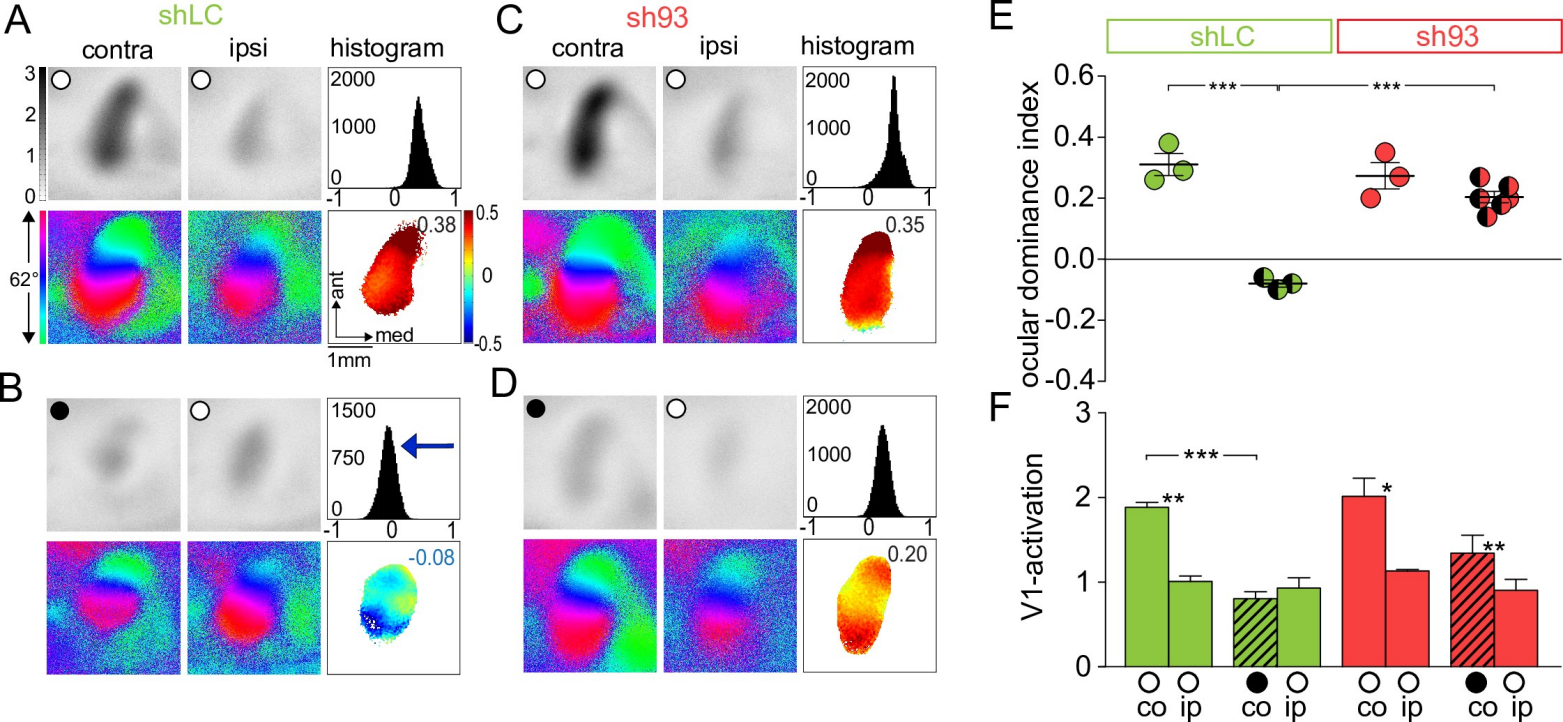
Knock-down of PSD-93 in the visual cortex phenocopied the PSD-93 KO effect: Juvenile ODP was absent in late critical period. (A-D) Optically imaged activity maps in V1 of WT mice with shLC (A, B) and with sh93 (C, D) during late CP (≥P28) before (A, C) and after 4 d of MD (B, D). Data displayed as in Fig 3. (E, F) Summary graph of ODI (E) and V1 activation (F). ○, open eyes; ●, deprived eyes. ODIs (E) and V1 activation (F) before (oo) and after (●o) 4 d MD in WT mice with shLC (green) and with sh93 (red). Data displayed as in Fig 3. *p < 0.05, **p < 0.01, ***p < 0.001. Underlying data for this figure can be found in S1 Data. ant, anterior; co, contralateral; contra, contralateral; CP, critical period; KO, knock-out; ip, ipsilateral; ipsi, ipsilateral; MD, monocular deprivation; ODI, ocular dominance index; ODP, ocular dominance plasticity; P, postnatal day; PSD, postsynaptic density; shLC, short hairpin RNA against luciferase; sh93, short hairpin RNA against PSD-93; V1, primary visual cortex; WT, wild-type.

PSD-93 cell-autonomously regulated silent synapse maturation (Fig 2). To test whether visual cortex–restricted deletion of PSD-93 expression was sufficient for precocious closure of the critical period for jODP, we delivered AAV-sh93 or AAV-shLC as control, into the visual cortex of WT P0–P1 mice [8]. V1 activities of non-deprived mice with AAV-sh93 or AAV-shLC were similar and dominated by visual inputs from the contralateral eye (Fig 4A and 4C); the ODI for both was also similar and positive (V1 activation shLC: contra versus ipsi, *p* < 0.01; V1 sh93: contra versus ipsi, *p* < 0.05; ODI: shLC no MD versus sh93 no MD, *p* = 0.55; Fig 4E and 4F). In AAV-shLC–expressing mice, 4 d MD induced an OD shift towards the non-deprived eye in the late critical period (≥P28) so that both eyes activated V1 more evenly and the ODI was reduced. In contrast, in AAV-sh93–expressing mice, no shift was induced in the late critical period and the deprived eye continued to dominate V1 (V1 activation after MD shLC: contra versus ipsi, *p* = 0.27; V1 after MD sh93: contra versus ipsi, *p* < 0.01; ODI after MD: shLC versus sh93, *p* < 0.001; Fig 4B and 4D–4F). Similar to PSD-93 KO mice, both V1 activity and retinotopic maps were not significantly altered: V1 activation (elevation: shLC versus sh93, *p* = 0.73; azimuth: shLC versus sh93, *p* = 0.79; S3 Fig) and retinotopic map quality (elevation: shLC versus sh93, *p* = 0.89; azimuth: shLC versus sh93, *p* = 0.72; S3 Fig) were indistinguishable between shLC and sh93 KD mice. Thus, visual cortex–restricted KD of PSD-93 phenocopied the effect of PSD-93 KO on jODP timing, indicating that PSD-93 expression in the visual cortex is required to prevent precocious critical period closure.

### Accelerated decrease of silent synapses in PSD-93 KO mice is mediated by precocious synaptic maturation

**Fig 5:**
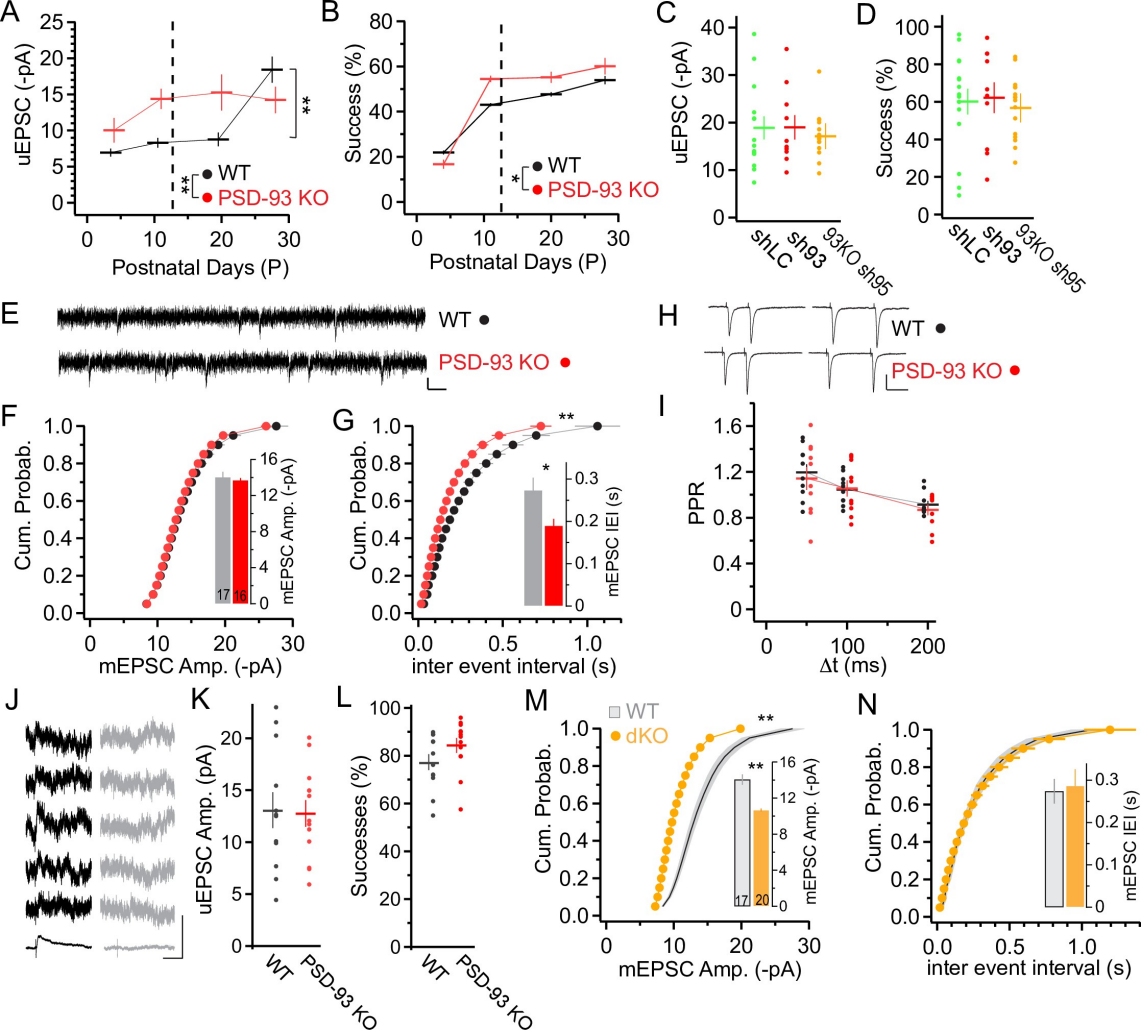
Accelerated maturation of silent synapses in PSD-93 KO mice: developmental time course of synaptic potency. (A, B) uEPSC (A) and success rate (B) of EPSCs evoked with minimal stimulation (recordings from Fig 1) in V1 slices of WT (black) and PSD-93 KO mice (red). Vertical dashed line illustrates time point of eye opening. Two-factor ANOVA for difference between genotypes and one-factor ANOVA with Tukey to test for differences between individual groups; *p < 0.05, **p < 0.01. Underlying data for this figure can be found in S1 Data. (C, D) Potency (C) and success rate (D) of EPSCs evoked with minimal stimulation from P28 WT mice, expressing shLC (green), sh93 (red), or PSD-93 KO with sh95 (orange) (recordings from Fig 2). Underlying data for this figure can be found in S1 Data. (E-G) mEPSC recordings with sample traces (E) of WT (top) or PSD-93 KO (bottom) and cumulative probability graph of mEPSC amplitude (F) and IEI (G) for WT (black) and PSD-93 KO (red) mice at P23 (P20–P26). Average mEPSC amplitude (F) and IEI (G) is illustrated in the inset. Number of layer 2/3 pyramidal neurons indicated in the foot of bar. KS test for equal distribution or *t* test for difference of means, *p < 0.05, **p < 0.01. Scale bar: 10 pA, 50 ms. Underlying data for this figure can be found in S1 Data. (H, I) PPR with different interstimulation intervals (Δt, 50 ms, 100 ms, 200 ms), with sample traces (H) for 50 ms and 100 ms for WT (H, top) and PSD-93 KO (H, bottom) at P24. Summary graph (I) with values for single neurons (dots) and average (horizontal line) for WT (black) and PSD-93 KO (red) mice at P25. Scale bar: 50 pA, 50 ms. Underlying data for this figure can be found in S1 Data. (J-L) Potency (K) and success rate (L) of NMDA receptor EPSCs (V_h_ = +40 mV) evoked with minimal stimulation from P24 WT or PSD-93 KO mice. To measure the potency, traces for successes for each cell were pooled and averaged, and the peak amplitude of the averaged trace measured and subtracted from the amplitude at the same time point of the averaged failure traces (J). Scale bar: 50 pA, 200 ms. Underlying data for this figure can be found in S1 Data. (M, N) mEPSC recordings plotted as cumulative probability graphs of mEPSC amplitude (M) and IEI (N) for WT (gray) or PSD-93/95 dKO (orange) mice at P23 (P20–P26). Average mEPSC amplitude (M) and IEI (N) are illustrated in the inset. Number of layer 2/3 pyramidal neurons indicated in the foot of bar. Data for WT are the same as in panels F and G. KS test for equal distribution or *t* test for difference of means, **p < 0.01. Underlying data for this figure can be found in S1 Data. dKO, double KO; EPSC, excitatory postsynaptic current; IEI, inter-event interval; KO, knock-out; KS, Kolmogorov-Smirnov; m, miniature; NMDA, N-methyl-D-aspartate; P, postnatal day; PPR, paired pulse ratio; PSD, postsynaptic density; shLC, short hairpin RNA against luciferase; sh93, short hairpin RNA against PSD-93; sh95, short hairpin RNA against PSD-95; uEPSC, unitary EPSC; V_h_, holding potential; V1, primary visual cortex; WT, wild-type.

Silent synapses mature (unsilence) by long-term synaptic potentiation (LTP)-driven incorporation of AMPARs [35–38]. However, the results on the developmental time course of silent synapses, especially in the PSD-93 KO mice, did not allow us to determine whether the decrease was due to the maturation of silent synapses or their elimination, two processes that likely occur competitively during experience-dependent cortical network refinement [39]. To resolve these two possibilities, we analyzed additional synaptic parameters from the minimal stimulation assay. In the cortex, one glutamatergic axon forms on average five synapses with a target pyramidal neuron [40]. Thus, with the maturation of silent synapses and the resulting decrease of the fraction of silent synapses, the amplitude of the unitary response will increase [27]. In both PSD-93 KO and WT mice, the amplitude of the successes (potency) increased during development (two-way ANOVA: *F*_3,120_ = 6.89, *p* < 0.001; Fig 5A), but the time course of this developmental increase was different between the two genotypes (two-way ANOVA: genotype, *F*_1,120_ = 4.453, *p* < 0.05; interaction, *F*_3,120_ = 4.332, *p* < 0.01). In WT mice, synaptic potency increased from P20 to P28 (*F*_7,120_ = 5.61, *p* < 0.01; WT: P20 versus P28, *p* < 0.01; Fig 5A). In contrast, in PSD-93 KO mice, it already reached the high level at P11, similar to that of WT mice at P28 (PSD-93 KO P11 versus WT P28, *p* = 0.52; Fig 5A). Furthermore, the success rate of the minimal AMPAR excitatory postsynaptic currents (EPSCs) increased during development (two-way ANOVA: *F*_3,127_ = 27.49, *p* < 0.001; Fig 5B), and was higher in PSD-93 KO mice than in WT mice (*F*_1,127_ = 4.054, *p* < 0.05; Fig 5B). We also assessed the potency and success rate in shLC, sh93, and dKO/KD neurons. At P28, both the potency and success rate were similar (potency: *F*_2,38_ = 0.22, *p* = 0.80; success: *F*_2,38_ = 0.15, *p* = 0.86, Fig 5C and 5D). Together, these results indicate that the decrease of the fraction of silent synapses is at least partially mediated by an increase in mature synapses in all WT, PSD-93–lacking, and PSD-93/95–lacking neurons, and that in PSD-93 KO mice, maturation already starts before eye opening, while in WT mice, it is restricted to the critical period after P20.

While the minimal stimulation assay of L4 to layer 2/3 pyramidal cells allowed us to assess the maturation state of synapses from single or few stimulated axons, it did not reveal whether loss of PSD-93 affected the total number of synaptic connections onto layer 2/3 pyramidal neurons. To address this question, we measured miniature (m) EPSCs from layer 2/3 pyramidal neurons at P24, a time point at which the difference of the fraction of silent synapses between WT and PSD-93 KO mice was high (Fig 1). The mEPSC amplitude distribution and its average value was similar between WT and PSD-93 KO mice (Kolmogorov-Smirnov [KS] test: *p* = 0.84; *t* = 0.532, *p* = 0.60; Fig 5F), indicating that the synaptic strength of individual AMPA receptor–positive synapses was similar. However, the mEPSC frequency was higher in PSD-93 KO mice than that in WT mice (KS test: *p* < 0.01; *t* = 2.65, *p* < 0.05; Fig 5G).

Although the mEPSC amplitude revealed no changes in the average AMPA receptor function per synapse sampled over all layer 2/3 pyramidal neuron synapses in PSD-93 KO mice, we had not yet ruled out a selective change in the L4 onto layer 2/3 pyramidal cell synapses. The substitution of Sr^2+^ for Ca^2+^ in the artificial cerebrospinal fluid desynchronizes synaptic vesicle release, so that individual quantal responses can be analyzed in the stimulated synaptic pathway to specifically assess quantal size for the L4 to layer 2/3 synapses [41]. The amplitude of these evoked mEPSCs was similar in WT and PSD-93 KO mice (KS test: *p* = 0.90; *t* = 0.29, *p* = 0.78; S4B and S4C Fig), indicating no contribution of the quantal response, such as synaptic potentiation, to the increase of the unitary response in PSD-93 KO mice. Notably, the amplitude of the evoked mEPSCs was similar to that of spontaneous mEPSCs (*t* = 0.055, WT mEPSC versus WT evoked mEPSC, *p* = 0.96), indicating that the quantal size of the L4-to-L2/3 synaptic connection is similar to that of the average connection onto L2/3 pyramidal neurons. Similar to the mEPSC inter-event intervals, the inter-event intervals of the evoked mEPSCs were smaller in PSD-93 KO compared with that of WT mice (WT, 355.9 ± 46.1 ms; PSD-93 KO, 221.6 ± 28.7 ms; *t* = 2.47, *p* < 0.05).

**Fig 6:**
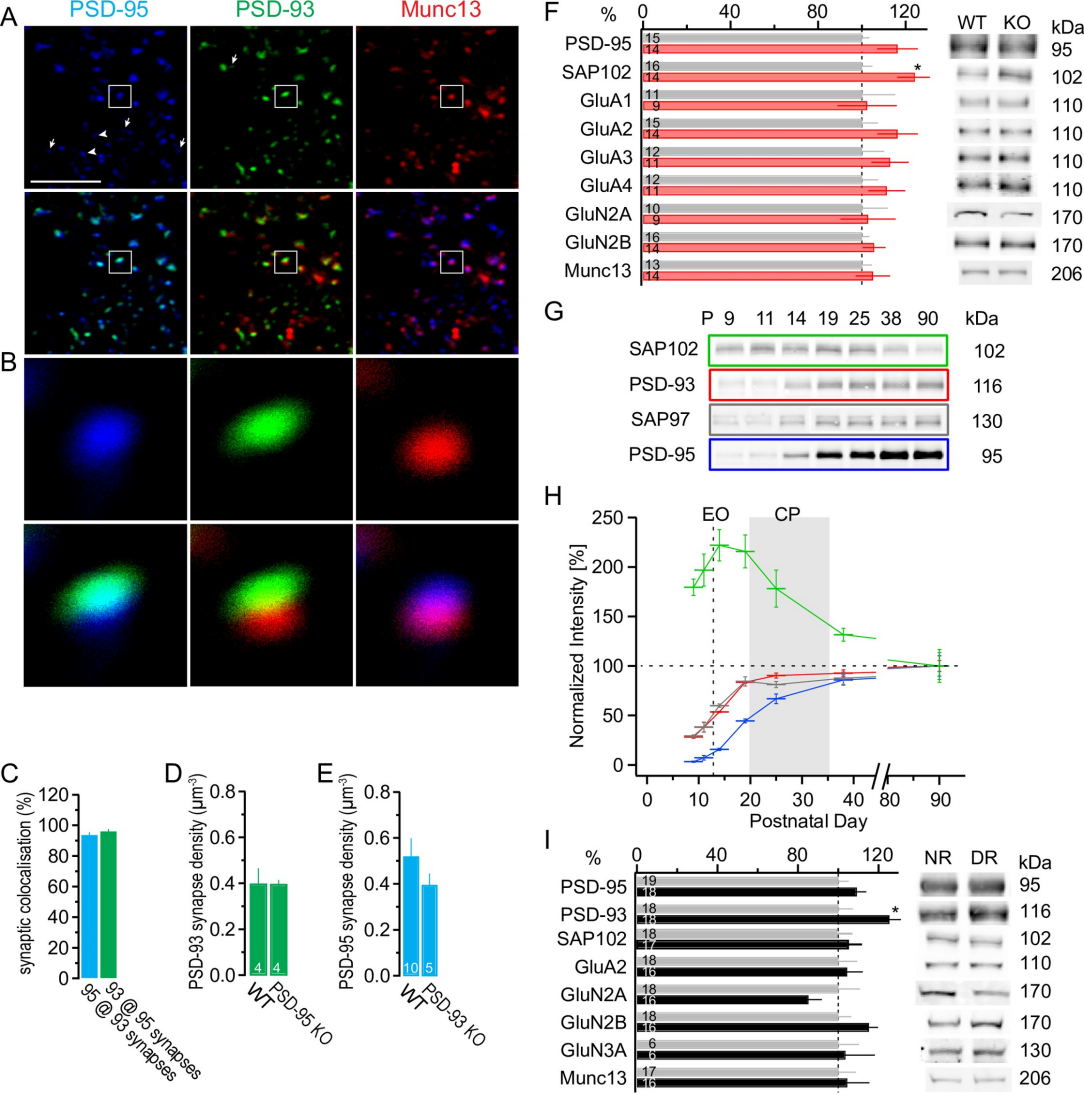
Synaptic and developmental profile of PSD-93 and PSD-95. (A, B) Immunofluorescence labeling of semi-thin sections with overview (A) and enlargement of boxed area (B) of mouse (P40) visual cortex for PSD-95 (blue), PSD-93 (green), and Munc13-1 (red). Upper panels illustrate fluorescence for single channels and lower panels for two channels, with PSD-95/PSD-93 (left), PSD-93/Munc13-1 (middle), and PSD-95/Munc13-1 (right). White arrowheads depict puncta with only one paralog colocalized with Munc13-1, and white arrows depict puncta of paralogs not colocalized with Munc13-1. Scale bar: 5 μm. (C-E) Quantification of synaptic colocalization (C) of PSD-95 in PSD-93/Munc13-1 positive puncta (blue) and of PSD-93 in PSD-95/Munc13-1 positive puncta (green). Synapse density, defined as puncta with PSD-93/Munc13-1 colocalization (D) in WT and PSD-95 KO mice. Similarly defined PSD-95 synapse density in WT and PSD-93 KO mice. Number of animals in foot of bar. See also S5 Fig. Underlying data for this figure can be found in S1 Data. (F) Protein levels in crude synaptosomes of the visual cortex in P28 WT (gray) or PSD-93 KO mice (red). Values of samples from each mouse were normalized to the average value of WT mice for each indicated protein on a western blot and presented as the relative amount compared with the WT band intensity. Sample bands for each protein and genotype are illustrated on the right. *t* test, *p < 0.05. (G-H) Developmental profile of synaptic proteins PSD-95 (blue), PSD-93 (red), SAP97 (gray), and SAP102 (green) from crude synaptosomal fractions from V1 of WT mice. Sample blots (G) are illustrated for the indicated proteins, and quantified protein levels, normalized to the adult levels at P90, are plotted against the postnatal day (P, H). *n* = 4–5 (mice). Values for PSD-95 are from a previous report [8]. Underlying data for this figure can be found in S1 Data. (I) Comparison of synaptic protein levels from crude synaptosomal fractions from DR (black) and NR (light gray) mice at P28. Protein levels were assessed as described in panel F. Number of mice is indicated in the foot of the bar. *t* test, *p < 0.05. Underlying data for this figure can be found in S1 Data. CP, critical period; DR, dark-reared; EO, eye opening; Glu, glutamate receptor subunit; KO, knock-out; Munc13-1, Mammalian uncoordinated 13–1; NR, normal-reared; P, postnatal day; PSD, postsynaptic density; SAP, synapse-associated protein; V1, primary visual cortex; WT, wild-type.

While mEPSC frequency is a measure for the number of AMPAR-positive synapses, it is not a specific assay and can be influenced by synaptic vesicle fusion propensity, often referred to as release probability. To test whether the increase of mEPSC frequency was solely due to the increased number of matured silent synapses, we performed additional experiments. We previously reported that the number of AMPARs and NMDARs was unaltered in the PSD fraction of adult PSD-93 KO mice [42]. It was thus unlikely that an increase in synapse number caused the increase in mEPSC frequency (see also Fig 6). To reveal whether changes in release probability contributed to the increase, we performed two additional electrophysiological assays. The paired pulse ratio of two shortly spaced synaptic responses is a sensitive measure of release probability [43,44]. At three different interstimulus time intervals, paired pulse ratios were similar in WT and PSD-93 KO mice (two-way ANOVA: time interval, *F*_2,59_ = 9.29, *p* < 0.01; genotype, *F*_1,59_ = 0.33, *p* = 0.57; Fig 5H and 5I). In the second test, we performed the use-dependent NMDAR blocking assay with the open channel NMDAR blocker MK-801. This assay is based on the progressive decrease of NMDAR-mediated EPSCs, which is directly influenced by the presynaptic release probability [45]. Consistent with the results in the paired pulse ratio measurement, the time course of NMDAR EPSC blockade in WT and PSD-93 KO mice was similar (two-way ANOVA: genotype, *F*_1,1040_ = 1.01, *p* = 0.32; sweep number, *F*_49,1040_ = 71.89, *p* < 0.01; interaction, *F*_49,1040_ = 0.29, *p* = 1; S4A Fig). Together, these additional electrophysiological results reveal that loss of PSD-93 did not significantly affect release probability, and the increase in mEPSC frequency was primarily mediated by the increased number of AMPAR-positive synapses, i.e., a lower fraction of silent synapses.

A previous study reported an increase in the ratio of AMPAR/NMDAR EPSCs in the cortex of PSD-93 KO mice [25]. These studies concluded that NMDAR function was selectively decreased in PSD-93 KO mice. We thus analyzed NMDAR EPSCs in L4 onto layer 2/3 pyramidal cell synapses in detail. The ratio of AMPAR/NMDAR EPSCs was increased in PSD-93 KO mice compared with that of WT mice (Mann-Whitney [MW] test: *p* < 0.01; S4F and S4G Fig). This change could be caused by an increase in AMPAR EPSC amplitudes, e.g., by an increase of AMPAR-positive synapses and/or a decrease in NMDAR EPSC amplitudes. To compare the size of NMDAR EPSCs between the genotypes, we measured their sizes by minimal stimulation that allows the comparison of absolute values across recordings from different slices. The NMDAR EPSC amplitudes in WT and PSD-93 KO mice were similar (*t* = 0.15, *p* = 0.88; Fig 5J and 5K), as were the success rates (*t* = 1.56, *p* = 0.13; Fig 5L). Together with the unchanged release probability, these results indicate a similar connectivity between individual L4 star pyramids and layer 2/3 pyramidal neurons in the two genotypes. In conclusion, silent synapses mature precociously in the visual cortex of PSD-93 KO mice, while synapse density and NMDAR EPSCs were not affected in the critical period.

### The number of AMPA receptor–positive synapses is unaltered in PSD-93/95 KO mice during the critical period

Because the mEPSC frequency provided a good estimate of the relative changes of AMPAR-positive synapses, we used this assay to test PSD-93/95 dKO mice. At P24, the mEPSC frequency was similar to that of WT mice (KS test: *p* = 0.24; *t* = 0.28, *p* = 0.78; Fig 5N), corroborating our result from the silent synapse measurement and indicating that in the dKO mice, the number of synapses and the fraction of silent synapses was similar to that of WT mice. However, in dKO mice, the mEPSC amplitude was reduced compared with WT mice (KS test: *p* < 0.01; *t* = 5.59, *p* < 0.01; Fig 5M). Thus, maturation of silent synapses lacking both paralogs resulted in a reduced number of AMPARs in individual synapses.

For comparison, we also assessed the AMPAR-positive synapses in PSD-95 KO mice. In the hippocampus of PSD-95 KO mice, the mEPSC amplitude is unchanged and the frequency reduced [24]. In visual cortical layer 2/3 pyramidal neurons, both the frequency and the amplitude were reduced compared with WT mice (KS test: amplitude, *p* < 0.01; frequency, *p* < 0.01; S4D and S4E Fig), indicating that the impairment of silent synapse maturation may cause the reduction in AMPAR numbers. Collectively, these results show that silent synapses mature in the absence of both PSD-93 and PSD-95, but PSD-95 is required for the normal mature functional state, shown by WT-like AMPAR content (mEPSC amplitude) in individual synapses. PSD-93 counterbalances PSD-95 to drive synapses in the mature state and thus regulates the experience-dependent component of the maturation.

### Coexpression of PSD-93 and PSD-95 in mouse visual cortex synapses

PSD-93 and PSD-95 are abundant proteins at glutamatergic synapses [46,47]. While some studies report a colocalization at single PSDs of rat brain or at about 90% synaptic puncta in primary neuron cultures [48], other studies report a distribution of PSD-93 and PSD-95 to different synapses in the CA1 region of the hippocampus [23,49]. To assess the potential colocalization of PSD-93 and PSD-95 in mouse visual cortex, we decorated semi-thin slices with antibodies against PSD-93, PSD-95, and the presynaptic active zone marker Mammalian uncoordinated 13–1 (Munc13-1) to specifically identify synaptic puncta [50]. Immunofluorescence of PSD-93 and PSD-95 was punctate and overlapped about 95% at puncta associated with a presynaptic marker (Fig 6A–6C), indicating that these two paralogs are predominantly colocalized at the same synapses. The specificity of the PSD-93 or PSD-95 labeling was validated with semi-thin slices from the corresponding KO mice (S5 Fig). While in PSD-95 KO mice, the signal for PSD-95 was diminished to background levels, in PSD-93 KO mice, some—but <10% of the number of puncta compared with WT or PSD-95 KO slices—synaptic puncta were still stained by the PSD-93 antibody. However, these puncta are not PSD-93, as no residual PSD-93 is detectable in western blots of cortical protein extracts [19,34,42]. Nevertheless, a small fraction of the PSD-93–positive puncta in WT slices might originate from antibody cross-reactivity without changing our overall conclusion of colocalization of PSD-93 and PSD-95 in individual visual cortical synapses.

The slice thickness of 500 nm minimizes confounding staining of synapses that may overlap in different layers in thick slices. Notably, some puncta were only labeled for Munc13-1 and thus likely represent inhibitory synapses. We assessed the density of excitatory synapses by counting the puncta with Munc13-1/PSD-95 colocalization or Munc13-1/PSD-93 colocalization. Consistent with the high degree of colocalization of PSD-93 and PSD-95, excitatory synapse density was similar, about 0.4 per μm^3^, for both approaches. In PSD-95 KO mice, the PSD-93–based synapse density was similar to that of WT mice (*p* = 0.99; Fig 6D). Similarly, in PSD-93 KO mice, the PSD-95–based synapse density was similar to that of WT mice (*p* = 0.095; Fig 6E). Thus, consistent with the electrophysiological assessments, the number of excitatory synapses was not changed in either PSD-93 or PSD-95 KO mice at P40.

To test for protein alterations in the synaptic composition of PSD-93 KO mice, we isolated crude synaptosomal fractions from the visual cortex of approximately P28 mice. The protein levels of PSD-95, Munc-13, and glutamate receptor subunits (Glu) were not significantly altered (Fig 6F). However, synapse-associated protein (SAP)102 levels were increased in PSD-93 KO mice (*t* = −2.60, *p* < 0.05; Fig 6F). Notably, in PSD-95 KO mice, the early expressed paralog SAP102 is also increased [8,51], indicating that in both single-KO mice, SAP102 levels were increased.

In the visual cortex, PSD-95 protein levels increase temporally in parallel to silent synapse maturation after eye opening [8]. To test whether PSD-93 levels are also correlated with silent synapse maturation, we measured the level of PSD-93 together with other PSD/synaptic proteins in crude synaptosomal fractions during normal standard cage rearing. Similar to PSD-95, PSD-93 protein levels increased from low levels before eye opening to plateau already at adult levels during the critical period (P38, two-factor ANOVA, age: *F*_6,57_ = 145, *p* < 0.001; Fig 6G and 6H). Similarly, SAP97 protein levels increased steeply after eye opening throughout the critical period. The developmental increase of PSD-93 was shifted to younger ages and thus increased relatively faster than PSD-95 (two-factor ANOVA, genotype: *F*_1,57_ = 120, *p* < 0.01; interaction age and genotype: *F*_6,57_ = 6.74, *p* < 0.01; Fig 6H). These results reveal that before eye opening, only small amounts of PSD-93 and PSD-95 are expressed in the visual cortex, further corroborating our result that the fraction of silent synapses at P4 was similar in WT, PSD-93 KO, and PSD-93/95 dKO mice (Figs 1 and S1).

The developmental profile of SAP102 was different. Its protein levels peaked after eye opening and progressively decreased during the critical period (Fig 6G and 6H). A group of proteins exhibited a similar developmental pattern, including GluN2B, GluN3A, and ras-related protein Rab3B (S6 Fig) [8], indicating a common role of these proteins in an immature state of synapses [42,52]. Another group of proteins that exhibited a similar developmental pattern as PSD-95, particularly a preferential increase during the critical period, included the voltage-gated potassium channel subunit (Kv)1.1 and the vesicular glutamate transporter (vGluT)1, while most other synaptic proteins increased to adult levels before the onset of the critical period (S6 Fig) [8].

Next, we tested whether the increase of PSD-93 and PSD-95 protein levels in the visual cortex were visual experience dependent. PSD-95 protein levels exhibited similar increases throughout development between DR and NR mice (*t* = −1.30: *p* = 0.20; Fig 6I), indicating that the developmental increase of PSD-95 protein levels is independent of visual experience. In contrast, PSD-93 protein levels were higher in DR mice than in NR mice (*t* = −2.32: *p* < 0.05; Fig 6I), revealing differences in the regulation of PSD-93 and PSD-95 protein levels by visual experience. Confirming previous results [53], dark rearing changed the NMDAR subunit composition towards a decreased GluN2A/GluN2B ratio (MW: NR, 1.06 ± 0.12 versus DR, 0.72.2 ± 0.051, *p* < 0.01). Other tested synaptic protein levels were similar in NR and DR mice. In DR PSD-93 KO mice, SAP102 protein levels were increased, whereas the GluN2A/GluN2B ratio was similar compared with that in NR WT mice (SAP102: t = −2.36, *p* < 0.05; GluN2A/GluN2B ratio: MW, *p* = 0.13; S6 Fig). As dark rearing did not affect silent synapse maturation in PSD-93 KO mice and it progressed faster (Fig 2), these results indicate that SAP102 protein levels are regulated by PSD-93 or PSD-95, while the GluN2A/GluN2B ratio is implicated in silent synapse maturation.

In summary, these results reveal that PSD-93 and PSD-95 are predominantly expressed together in individual excitatory synapses in the visual cortex. Despite the halted silent synapse maturation in the visual cortex in DR mice (Fig 2) [27], PSD-95 protein levels increased. This result seemingly disconnects the PSD-95–level increase from silent synapse maturation and hence the well-established correlation of PSD-95 protein levels and excitatory synaptic strength [23,54,55]. However, the increase of PSD-93 and its inhibiting function in silent synapse maturation offers a potential mechanism: during normal development, PSD-93 prevents PSD-95–promoted silent synapse maturation by counteracting PSD-95.

### PSD-93 or PSD-95 gain of function oppositely governs the time course of silent synapse maturation

**Fig 7:**
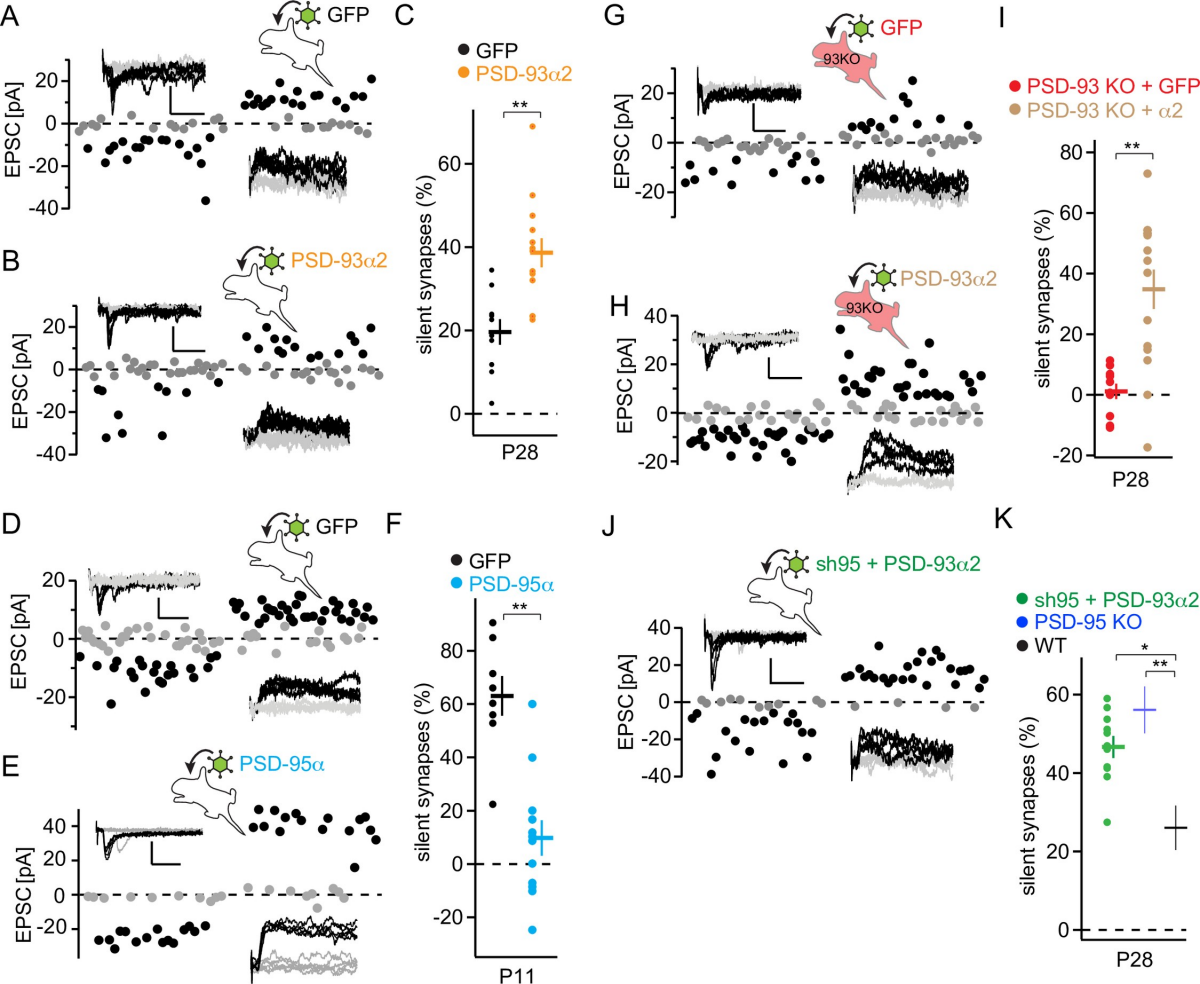
PSD-93α2 opposes PSD-95 function in the same molecular pathway. (A, B, D, E, G, H, J) Sample traces of V1 layer 2/3 pyramidal neuron EPSCs with minimal stimulation of P28 WT mice with AAV-GFP (A), with AAV-PSD-93α2 (B), with AAV-sh95 + PSD-93α2 (J), P11 WT mice with AAV-GFP (D), with AAV-PSD-95α (E), P28 PSD-93 KO mice with AAV-GFP (G) and with AAV-PSD-93α2 (H), with sample traces (inset) and analysis of the peak values of AMPA receptor EPSCs (downward deflection) or composite glutamate receptor EPSCs (upward deflection) of successes (black) and failures (gray) of individual EPSCs. Scale bar: 20 ms and 25 pA. Schematic drawing of stereotactic injection of indicated AAV into the visual cortex of P0 mice. (C, F, I, K) Summary graphs of the fraction of silent synapses of indicated manipulation (color code). Dots represent value of single neuron. Values for PSD-95 KO and WT mice (K) were obtained from Fig 1. *p < 0.05; **p < 0.01. Underlying data for this figure can be found in S1 Data. AAV, adeno-associated viral vector; AMPA, α-amino-3-hydroxy-5-methyl-4-isoxazole propionic acid; EPSC, excitatory postsynaptic current; GFP, green fluorescent protein; KO, knock-out; P, postnatal day; PSD, postsynaptic density; sh95, short hairpin RNA against PSD-95; V1, primary visual cortex; WT, wild-type.

Our results are based so far on PSD-93 or PSD-95 loss of function. To test whether (1) the converse, the gain of function, also governs the time course of silent synapse maturation and (2) which of the six PSD-93 and two PSD-95 N-terminal isoforms can mediate this, we chose the following isoforms based on previous reports. In organotypic hippocampal slice cultures, PSD-95α overexpression accelerates silent synapse maturation [56], whereas PSD-93α2 overexpression reduces AMPAR EPSCs [19]. For the in vivo manipulation, we expressed the PSD-95α and PSD-93α2 isoforms with AAVs and stereotaxically injected them at P0–P1 into the visual cortex of WT mice. Overexpression of PSD-93α2 increased the fraction of silent synapses at P28 compared with green fluorescent protein (GFP)-expressing pyramidal neurons (*t* = 4.02, *p* < 0.01; Fig 7A–7C), indicating that a gain of function of the α2 isoform of PSD-93 is sufficient to oppose silent synapse maturation.

In contrast, overexpression of PSD-95α decreased the fraction of silent synapses at P11 compared with GFP-expressing pyramidal neurons (*t* = 5.32, *p* < 0.01; Fig 7D–7F), indicating that expressing PSD-95α before eye opening is sufficient to induce silent synapse maturation to reach to the adult level precociously. Thus, gain of PSD-93 or PSD-95 function affected silent synapse maturation in opposite directions, echoing the results from the loss of function approaches.

Using a molecular replacement approach, we expressed PSD-93α2 in the background of PSD-93 KO. In PSD-93α2–expressing neurons at P28, the fraction of silent synapses was higher compared with that in GFP-expressing neurons (*t* = 4.84, *p* < 0.01; Fig 7G–7I). Thus, the PSD-93α2 isoform alone was sufficient to prevent precocious silent synapse maturation in PSD-93 KO mice.

The increase of PSD-93 protein levels in DR mice indicated that a gain of PSD-93 function opposes silent synapse maturation (Fig 6). Furthermore, PSD-93 and PSD-95 are primarily expressed in the same synapses (Fig 6). Together, these results imply that PSD-93 opposes PSD-95 during silent synapse maturation in the same functional pathway. To directly test this hypothesis, we performed an epistasis experiment, combining PSD-95 KD with PSD-93α2 overexpression. The rationale was that if both proteins function through independent mechanistic pathways, the effects of the combined interrogation should be additive. However, in sh95 + PSD-93α2–expressing neurons at P28, the fraction of silent synapses was not further increased compared with that of PSD-95 KO mice, but was higher compared with that of WT mice (*F*_2,42_ = 8.493, *p* < 0.01; sh95 + PSD-93α2 versus PSD-95 KO, *p* = 0.48; sh95 + PSD-93α2 versus WT, *p* < 0.01; Fig 7J and 7K). A lack of increase was unlikely caused by the ceiling effect at about 50% silent synapses, considering the fraction of silent synapses at 80% in P4 mice (Fig 1). Thus, PSD-93 opposes the PSD-95–dependent maturation of silent synapses through the same mechanistic pathway.

### Visual acuity is impaired in PSD-93/95 dKO, but not in PSD-93 KO mice

**Fig 8:**
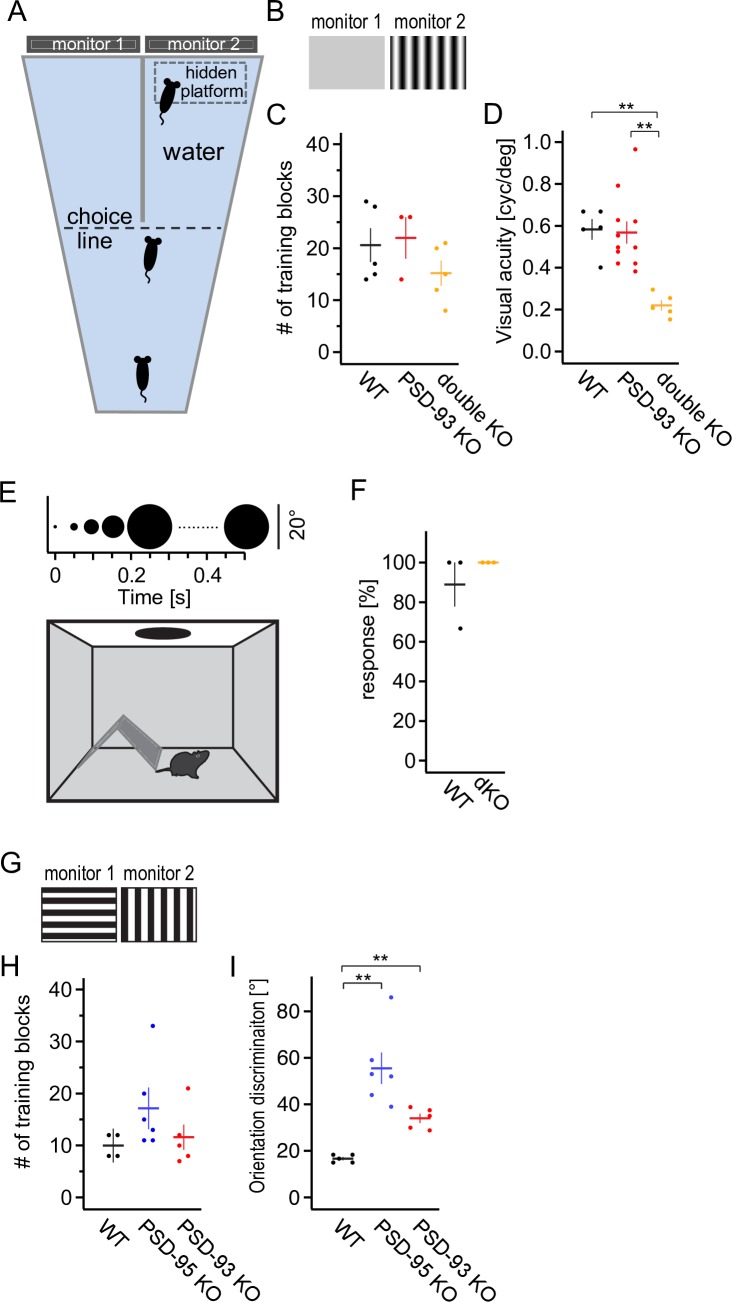
Acquired, but not innate, visual capabilities are impaired by loss of the DLG-MAGUK paralogs. (A, B) Scheme of the VWT apparatus (A) showing the trapezoid water-filled pool, the midline divider, choice line, and the monitors on which visual stimuli are projected. For measuring visual acuity, mice were trained to swim towards the sinusoidal vertical grating (B; rewarded stimulus). For measuring orientation discrimination, mice were trained to swim towards square-wave vertical grating (G; rewarded stimulus), and the initial horizontal grating was turned by 5° per trial to approach the vertical orientation. (C, H) Number of training blocks to learn the visual acuity procedure (C) for WT, PSD-93 KO, and PSD-93/95 dKO mice or orientation discrimination procedure (H) for WT, PSD-95 KO, or PSD-93 KO mice. Values of individual mice are presented as dots and mean as horizontal line. Underlying data for this figure can be found in S1 Data. (D, I) Visual acuity (D) or orientation discrimination (G) threshold for indicated mouse groups. **p < 0.01. Underlying data for this figure can be found in S1 Data. (E) Schematic representation of the looming procedure with a mouse in a dark arena with a shelter hut and a bright ceiling, on which the dark looming spot is presented. Time course of looming spot presentation is illustrated at the top. (F) The fraction of responses of three consecutive trials (24 h apart) is plotted against WT and PSD-93/95 dKO mice. Values of individual mice are presented as dots and mean as horizontal line. **p < 0.01. Underlying data for this figure can be found in S1 Data. dKO, double KO; DLG, disc-large; KO, knock-out; MAGUK, membrane-associated guanylate kinase; PSD, postsynaptic density; VWT, visual water task; WT, wild-type.

Our results so far reveal opposing functions of PSD-93 and PSD-95 in both silent synapse maturation and the timing of the critical period for jODP (Figs 1, 3 and 7) [8]. We next tested whether the balanced function of PSD-95 and PSD-93 on silent synapse maturation is required for neural network refinement during critical periods to optimize visual capabilities. We used the visual water task (VWT), a visual discrimination task based on reinforcement learning [57]. In this test, mice were trained in a trapezoid basin filled with shallow water (Fig 8A) to distinguish a vertical sine wave grating from an isoluminant gray stimulus to measure visual acuity (Fig 8B). The sinusoidal grating was randomly presented on either the left or the right monitor at the wide end of the pool and rewarded with an invisible escape platform below the water surface to enforce swimming towards the stimulus. After the mice learned to swim to the rewarded stimulus, the spatial frequency of the grating was gradually increased to test the mice’s visual acuity limit. Visual acuity is not altered in PSD-95 KO mice [8]. In WT, PSD-93 KO and PSD-93/95 dKO mice, the number of training blocks required to learn the task was similar between the three genotypes (Mantel-Cox; χ^2^(2) = 2.92, *p* = 0.23; Fig 8C), indicating that learning of the discrimination task was not significantly compromised in these mice. PSD-93 KO mice had a similar visual acuity as WT mice (*F*_2,20_ = 11.49, *p* < 0.01; WT versus PSD-93 KO, *p* = 0.98; Fig 8D). In PSD-93/95 dKO mice, however, visual capabilities were severely compromised (WT versus dKO, *p* < 0.01; Fig 8D). These results reveal that while silent synapses mature in the absence of PSD-93 and PSD-95, the refinement to acquire and improve vision critically depends on PSD-93 or PSD-95, a result consistent with the mechanistic difference in silent synapse maturation in the absence of the two paralogs compared with WT mice (Figs 2 and 5).

Notably, this was a sensory impairment, as the dKO mice learned the task similarly to WT mice (Fig 8C), indicating that learning per se was not significantly impaired in the dKO mice, but rather, their visual acuity was impaired. As for voluntary physical exercise, dKO mice exhibited greatly reduced running wheel use and rearing in the cylinder test (run: *t* = 7.58, *p* < 0.01; cylinder: *t* = 3.72, *p* < 0.01; S7 Fig). Despite this, these dKO mice were not significantly compromised in performing the reinforced exercise in the VWT.

To test whether the visual cortex of PSD-93/95 dKO mice was similarly organized to that of WT mice, we visualized V1 activity and retinotopic maps using intrinsic signal optical imaging. We stimulated the contralateral eye with either horizontally or vertically moving bars (S8A and S8D Fig). V1 activation for elevation (WT versus dKO, *p* = 0.051; S8G Fig) and retinotopic map quality (map scatter) (elevation: WT versus dKO, *p* = 0.11; azimuth: WT versus dKO, *p* = 0.39; S8H and S8J Fig) were similar in dKO and WT mice. However, V1 activation for azimuth was slightly increased in dKO mice (WT versus dKO, *p* < 0.05; S8I Fig). Collectively, these results indicate that basic visual activation of V1 was not severely altered in the absence of PSD-93 and PSD-95.

The expression of both PSD-93 and PSD-95 in the visual cortex increased during the critical period and was rather low before eye opening (Fig 6). This expression time course indicates a specific role in developmental plasticity and neural network refinement during the critical period. To test this hypothesis, we analyzed an innate behavior, the defensive response to looming visual stimuli (Fig 8E) [58]. Both WT and dKO mice responded similarly to the looming visual stimuli (*t* = −1; *p* = 0.42; Fig 8F).

Together, these and our previous results reveal that in both single PSD-93 KO and PSD-95 KO mice, visual acuity is similar to that of WT mice [8]. In contrast, visual acuity was severely impaired in the dKO mice, while their innate response to a looming object was similar to WT mice.

### Orientation discrimination is impaired in PSD-93 and PSD-95 KO mice

Visual information is segregated into distinct features and represented in the visual cortex in feature-tuned neurons [59]. The percept of the original image is computed by feature integration through interconnected brain areas. To assess the role of silent synapse-based neural network refinement for improving visual perception, we used a variant of the VWT. We trained mice on discriminating gratings of different orientations. Initially, animals learned to discriminate vertical from horizontal gratings (Fig 8G). Again, the rewarded stimulus was presented randomly on the left or right monitor to avoid location bias. After learning the task, in which WT, PSD-95 KO, and PSD-93 KO mice needed a similar number of training blocks (Mantel-Cox; χ^2^(2) = 2.76, *p* = 0.25; Fig 8H), the orientation of the nonrewarded stimulus was successively altered in 5° steps towards the orientation of the rewarded stimulus. The orientation discrimination was reached once the success rate of the mice to make the correct choice fell below 70%, a criterion also used in the task for visual acuity. Both PSD-93 and PSD-95 KO mice exhibited impaired orientation discrimination and required a larger angular difference to perceive the rewarded stripes correctly (*F*_2,15_ = 18.59, *p* < 0.01; PSD-95 KO versus WT, *p* < 0.01; PSD-93 KO versus WT, *p* < 0.05; Fig 8H and 8I). These results reveal that both PSD-93 and PSD-95 are required to achieve optimal orientation discrimination abilities, likely by the cooperative function of these proteins to achieve the proper pace of silent synapse-based neural network refinement.

## Discussion

Two genome duplications in evolution expanded the number of paralogs from the ancestor genes to increase the complexity and protein repertoire in synapses of vertebrates, including the postsynaptic signaling scaffolds of the disc-large (DLG)-membrane-associated guanylate kinase (MAGUK) family of proteins [2]. Here, we show that, contrary to the general belief of redundant functions of paralogs, the DLG-MAGUK paralogs PSD-93 and PSD-95 opposingly regulated silent synapse maturation in the visual cortex during critical periods. The maturation of silent synapses was instructive for critical period plasticity and governed the refinement process of visual cortex networks to optimize visual perception, but not visual acuity, thus uncovering a mechanistic difference in the development of two visual functions. This finding contrasts with the conceptual framework of local inhibition to govern critical period plasticity, but establishes an essential link between silent synapse-based refinement of synaptic connectivity between principal neurons and critical period plasticity. Given the genetic association of PSD-95, PSD-93, and their binding partners with neurodevelopmental disorders, their critical role in sensory development with the delicate functional balance in pacing silent synapse maturation might hint to pathomechanisms of mental disorders, which clinically manifest often after critical periods of typical disorder-relevant cortical functions.

### The opposing roles of PSD-93 and PSD-95 in silent synapse maturation

The DLG-MAGUKs, including the paralogs PSD-93 and PSD-95, constitute signaling scaffolds of the PSD, which govern receptor signaling events in the PSD by assembling receptors with signaling enzymes and the effector proteins [2,60,61]. Similar to other paralogs, the DLG-MAGUK paralogs and their isoforms diversified from their ancestor gene *Dlg* in invertebrates to specialize for the complex demands in the vertebrate nervous system [3,19,54]. Among these, the palmitoylated α-isoforms regulate the number of synaptic AMPA receptors [23,54]. However, here, we show that PSD-93α2 inhibited PSD-95α in promoting synaptic AMPA receptor incorporation. More specifically, their cooperative function primarily regulates the unsilencing of AMPA silent synapses and not AMPAR numbers per synapse in general. These conclusions are based on the following results: the opposite phenotypes on silent synapse maturation in the respective KO mice (Figs 1 and 3) [8], the opposite phenotypes with gain of function by overexpression of PSD-95α versus PSD-93α2 (Fig 7), and loss and gain of PSD-95 or PSD-93 function affects primarily mEPSC frequency, rather than mEPSC amplitude (Figs 5 and S4) [19,23,24,26,56].

Furthermore, we show that PSD-93 and PSD-95 largely colocalize in the same synapse (Fig 6), as well as, in an epistasis test by combining PSD-95 KD with PSD-93α2 overexpression, the effect of increasing the fraction of silent synapses was not additive (Fig 7). These results support a model in which PSD-95α promotes the unsilencing of immature nascent synapses during developmental critical periods. The unsilenced mature state is maintained by the continuous presence of PSD-95, as loss of PSD-95 after maturation reinstates juvenile numbers of silent synapses [8]. PSD-93α2 inhibits this maturation by directly competing with PSD-95 in the same synapse. Notably, in DR mice, PSD-95 levels increased similarly to NR mice, but silent synapse levels remained high (Figs 2 and 6). In contrast, in DR mice, PSD-93 protein levels were increased above that in NR mice, a result supporting its active role in inhibiting PSD-95’s promoting effect on silent synapse maturation by direct competition. Similarly, PSD-93 protein levels increased earlier than PSD-95 protein levels after eye opening (Fig 6). Thus, the concerted expression of PSD-93 is critical to pace the maturation of silent synapses, and its levels are primarily adjusted during experience-dependent maturation.

Are PSD-93 and PSD-95 slots for AMPARs, as earlier reports suggested [62–65]? For PSD-93, our results are inconsistent with such a function, as with loss of PSD-93, synaptic AMPAR numbers in unsilenced synapses increased (Figs 1 and 2). Similarly, for PSD-95, such a function is unlikely, based on results both from this study and previous reports. In DR mice, PSD-95 protein levels increased, while the fraction of silent synapses remained high (Fig 6). This result seems at odds with the well-established correlation of PSD-95 protein levels and excitatory synaptic strength [23,54]. However, this result is in agreement with our model of opposing functions of PSD-93 and PSD-95, in which the balanced action of both proteins determines silent synapse maturation. Furthermore, in PSD-93/95 dKO mice, the fraction of silent synapses decreased with a similar pace after eye opening until late critical period, as in WT mice (Fig 2). Thus, just for the process of AMPAR synaptic incorporation, neither PSD-93 nor PSD-95 was required during this developmental time window. However, in the absence of these two paralogs, the mechanism of unsilencing differed and visual acuity was impaired, potentially by disrupting synaptic integrity or by differences in the synaptic state [42,63].

A previous report shows that the loss of PSD-93 and PSD-95 in the hippocampus produces additive decreases in AMPAR EPSCs [23]. We think that the apparent difference in result is primarily due to the different developmental time points studied. Given the mechanistic difference in silent synapse maturation between WT and PSD-93/95 dKO mice, our results do not exclude that the trajectories of silent synapse maturation between WT and PSD-93/95 dKO mice separate at a later developmental time point, and together with the reduced quantal size in PSD-93/95 dKO mice, result in reduced AMPAR EPSCs, as reported previously (Fig 5) [23].

Finally, LTP is enhanced in PSD-95 KO mice [24,26,66]. This result appears inconsistent with a slot function of PSD-95 but is consistent with PSD-95 regulating silent synapse maturation, as in PSD-95 KO mice, more silent synapses exist, which likely serve as substrates for LTP, lower the induction threshold, and increase LTP magnitude [26,35–38]. Collectively, these results are inconsistent with an AMPAR slot function of the DLG-MAGUKs. It should be noted that the DLG-MAGUKs execute functions additional to AMPAR regulation, as they outnumber AMPARs in the PSD more than 3-fold, they bind to other receptors and cell adhesion proteins, and loss of their function results in severe defects of the synaptic structure [63,67–69]. Therefore, interpretations of the results of multiple KO mice are complex. Importantly, loss of PSD-95 resulted in a 100% penetrant phenotype of loss of experience-dependent maturation of silent synapses after eye opening (Fig 1), so the interpretation of the results here were not complicated by redundancy or compensatory mechanisms, as the other DLG-MAGUK isoform, SAP97α, with a similar function to PSD-95α, is not expressed in detectable amounts [8,54].

SAP102 protein levels peaked at eye opening in the visual cortex and progressively decreased, while PSD-93 and PSD-95 protein levels increased (Fig 6). In both PSD-93 and PSD-95 KO mice, SAP102 protein levels are higher (Fig 6) [42,51]. These results are consistent with SAP102’s function as a signaling scaffold in regulating early synapse development and with SAP102 being replaced by PSD-93 and PSD-95 once synapses mature. Furthermore, because SAP102 protein levels are increased in both PSD-93 and PSD-95 KO mice, SAP102 is unlikely to account for the opposite effect on silent synapse maturation in PSD-93 and PSD-95 KO mice (Figs 1 and 2). This notion is further supported by previous reports showing a function of SAP102 in early development, primarily on NMDAR function and with limited effect on silent synapse numbers [52,70,71].

Our results support a direct competition of PSD-93 and PSD-95 in single synapses to either inhibit or promote silent synapse maturation, respectively. Both proteins are part of a big protein complex of the PSD, while SAP102, in contrast, is part of another, smaller protein complex [72,73]. These two complexes might represent different functional transmission sites in the synapse. Based on the developmental profile of SAP102 versus PSD-93 and -95 (Fig 6), the abundance of either complex changes during development from relatively more SAP102 complexes to relatively more PSD-93/95 complexes. The ratio of PSD-93 and -95 will then govern the stable incorporation of AMPARs. Our results show that dark rearing increases PSD-93 but does not affect the protein levels of PSD-95 (Fig 6). Furthermore, eye opening induces the translocation of PSD-95 into dendritic compartments [74]. Thus, both proteins are regulated by visual activity: the protein levels of PSD-93 and the dendritic localization of PSD-95. Consequently, the ratio of both proteins in the synapse might be controlled by both mechanisms.

The DLG-MAGUKs act as signaling scaffolds to transduce a receptor signal through signaling proteins onto effectors [60,61]. To exert the opposing function, PSD-93 and -95 may interact with different receptors, signaling proteins and/or effectors. Consistent with this hypothesis, PSD-95 binds directly to the signaling proteins striatal enriched protein tyrosine phosphatase (STEP)_61_ and Rous sarcoma virus oncogene tyrosine kinase family proteins Src, Lyn, and Yes, while PSD-93 associates preferentially with the Src family kinase Fyn [75–77]. Future studies will need to reveal whether these or other proteins contribute to the opposing function of the two signaling scaffolds PSD-93 and -95.

### The role of silent synapses in critical period plasticity

Critical periods are time windows of heightened neuronal plasticity, during which intrinsically connected cortical neural networks are refined by experience to optimize their functional output [9,10]. This plasticity is sharpened by local inhibitory circuits [78–81] and is limited by so-called plasticity brakes [82–86]. However, instructive changes in neural networks occur largely at excitatory synapses on principal neurons, which form the “memory engram” of refinements [87–89]. During early cortex maturation, silent synapses are abundant [35,38,90] and serve as synaptic substrates for experience-dependent neural network refinements [27,91,92]. We previously hypothesized a link between the instructive process of silent synapse-based cortical network refinement and the duration of critical periods [8]. In support of our hypothesis, we found that accelerated silent synapse maturation in PSD-93 KO mice precociously terminated the critical period of the juvenile form of ODP in the visual cortex (Fig 3). Together with our previous observations that lack of silent synapse maturation prevents the closure of the critical period [8], our current results reveal both a forward and a reverse correlation between progressive silent synapse maturation and the closure of the critical period. Notably, if silent synapses are reinstated after the closure of the critical period by knocking down PSD-95 in the adult visual cortex, both juvenile numbers of silent synapses and juvenile-like ODP are reinstated [8]. Furthermore, both silent synapse maturation and the duration of the critical period are extended by dark rearing (Figs 2 and 3) [27,93,94]. Further supporting our conceptual model of silent synapse–based critical period closure, dark rearing had no effect on silent synapse maturation in PSD-93 KO mice that matured precociously and concurrently terminated the critical period earlier (Figs 2 and 3). The strict correlation between the time course of silent synapse maturation and the duration of the juvenile form of ODP implies that silent synapses serve as the instructive substrates for refinement during critical periods: once they decline, critical periods end.

A similarly strict correlation is not consistently detected for the proposed inhibitory tone-based ending of the critical period. First, in PSD-95 KO mice, the inhibitory tone of parvalbumin-positive interneurons in V1 developed similarly to that in WT mice [8]. Nevertheless, jODP persisted lifelong in PSD-95 KO mice, indicating that a low inhibitory tone is not a prerequisite for jODP. Second, pharmacological increase of the inhibitory tone in PSD-95 KO mice in vivo does not prevent ODP [8]. Third, some studies detect an effect of dark rearing on γ-aminobutyric acid (GABA)-mediated transmission [95], while others do not [96]. Fourth, visual experience–dependent increases in brain-derived neurotrophic factor (BDNF) expression are prevented by dark rearing [97]. While transgenic expression of BDNF normalizes the delayed maturation of GABAergic inhibition by dark rearing and the closure of critical periods [94], BDNF is also critical for maturing silent synapses [37]. Thus, it is not clear whether the BDNF effect on critical periods was through inhibitory neurons or silent synapses. More likely, local inhibition and silent synapses act in concert to refine excitatory synapses of principal neurons, but the decline of silent synapses marks the end of the critical period.

### Dissociable functions of silent synapse maturation in visual acuity and perception

When assessing how silent synapse maturation affects vision, we observed dissociable consequences on different aspects of vision (Fig 8). First, loss of PSD-93 or PSD-95, resulting in either accelerated or impaired silent synapse maturation, respectively, did not impair visual acuity. However, loss of both PSD-93 and PSD-95, resulting paradoxically in a time course of silent synapse maturation more similar to WT mice, impaired visual acuity. Second, orientation discrimination was impaired in both PSD-93 and PSD-95 KO mice, i.e., independent of whether silent synapse maturation was accelerated or impaired. These results indicate that visual acuity is not dependent on proper silent synapse maturation, while visual perception is. Importantly, both visual acuity and orientation discrimination were tested with a similar reinforcement training (VWT), indicating that the selective impairment of orientation discrimination is likely based on perceptual impairments rather than other cortical functions, including learning and decision-making. Consistent with our results, previous studies in mice show that dark rearing, and thus preventing silent synapse maturation, does not prevent the maturation of visual acuity [33,98]. In contrast, binocular matching of orientation tuning and binocular vision require visual experience during critical periods [28,99–101]. Thus far, the cellular mechanisms for developing these features remained elusive. Our results now start to unravel the mechanistic differences. Silent synapses represent synaptic opportunities, which are consolidated into mature synaptic connections between principal neurons through experience-dependent processes [27,91,92]. We extend this concept by showing that a properly paced maturation is required to refine network function for receptive field integration to perceive orientation differences properly (Fig 8). The underlying mechanism of this silent synapse–based refinement is likely to alter the synaptic connection pattern and thus only occurs during critical periods when silent synapses are abundant. Which connection matures and is consolidated is of utmost importance, as it will impact the overall visual performance. As such, the maturation process is finely regulated from multiple angles, exemplified by the balanced cooperated regulations between PSD-93 and PSD-95. As a consequence, in both PSD-93 and PSD-95 KO mice, the regulation of silent synapse maturation is impaired, and the nonrefined synaptic connection pattern results in impaired orientation discrimination.

### The role of the glutamate receptor complex in neurodevelopmental disorders

The opposing function of PSD-93 and PSD-95 in regulating silent synapse maturation fundamentally changes the current conceptual framework of scaffolding proteins in glutamate receptor complexes. The DLG-MAGUKs, SAP90/PSD-95–associated proteins (SAPAPs), and Src homology domain and multiple Ankyrin repeat domains proteins (Shank) form a three-layered protein network attached to glutamate receptors and cell adhesion proteins [63,102–106]. Mutations in the genes of several components of this complex, especially Shank3 and Neuroligin 3 and 4, are linked to several genetic-associated neurodevelopmental disorders, in particular, ASD and schizophrenia [107–110]. PSD-95 is associated with a specific ASD-related syndrome, Williams syndrome [20,111]. Remarkably, in affected individuals, the developmental improvement of orientation discrimination, but not all other visual features, is halted at an immature childhood level, echoing our findings in PSD-95 KO mice [112]. Although it has long been known that components of the glutamate receptor complex and its syndrome-associated paralogs are critical for developmental glutamatergic synapse maturation [113,114], its role in developmental silent synapse maturation remains elusive. So far, a role in silent synapse maturation has been reported only for SAPAP3, Shank2, PSD-93, and PSD-95 (Fig 1) [8,70,115,116].

Critical periods for language skills and social interactions coincide with the typical onset of ASD in children, and critical periods for higher cognitive functions precede the typical time of clinical manifestation of schizophrenia in young adults. Furthermore, besides the characteristic symptoms of impaired social and language skills in autism and cognitive deficits in schizophrenia, sensory defects are also typical [12–14]. Thus, it is conceivable that scaffolding components of the glutamate receptor complex more generally contribute to silent synapse maturation and/or neural network refinement, and that this process is also critical for the pathogenesis of these neurodevelopmental disorders. As such, the visual perception defect as a consequence of PSD-93 or PSD-95–based developmental impairments can serve as a proxy what may go wrong in other cortical areas, as well. Consistent with this notion, we found similar maturational defects of silent synapses in the nucleus accumbens, hippocampus, and mPFC (Fig 1) [70]. Furthermore, cognitive impairments reminiscent of defects in ASD or schizophrenia were described in PSD-93 and -95 KO mice [3,117].

The involvement of PSD-93 in neurodevelopmental disorders might not be limited to the familial forms but also extend to idiopathic forms, because newly generated mutations causing schizophrenia are associated with *dlg2*, the gene for PSD-93, which appears to be highly susceptible for spontaneous somatic mutations [20–22]. In terms of the direction of synaptic alterations, be it precocious maturation or lack of maturation, future studies need to focus on whether PSD-93– or PSD-95–associated complexes are impaired to predict the direction of the maturational defects that cause alterations during neurodevelopment.

## Materials and methods

### Ethics statement

Experiments were approved by the Institutional Animal Care and Use Committee (IACUC) of the University of Pittsburgh (#18063191 and 2) and the Lower Saxony State Office for Consumer Protection and Food Safety.

### Mice

PSD-93 KO or PSD-95 KO mice and littermate controls were generated from heterozygous breeding pairs from a mixed 129SV/C57Bl6 background [8,34,70,118]. PSD-93/95 dKO mice were generated from PSD-93 KO and PSD-95 heterozygous breeding pairs. Mice were group housed, two to five per standard cage (33 cm × 17 cm), under a 12-h light/dark cycle with controlled temperature and humidity and were provided food and water ad libitum. All procedures were performed during the light cycle. For dark rearing, pregnant females were transferred to standard cages placed into a light-tight Scantainer (Scanbur Technology, Denmark) in a completely light-tight darkroom, with food and cage changes performed under red light illumination.

### Visual cortex slice preparation

Mice were killed under isoflurane anesthesia by decapitation. Coronal visual cortical, medial prefrontal cortical, or hippocampal slices (300 μm) of different age groups (P4 ± 1, P11 ± 1, P20 ± 1, P25 ± 4, P28 ± 2) of mice of either sex were sliced with a vibratome in ice-cold sucrose (in mM: sucrose 168, NaCl 25, KCl 1.9, MgSO_4_ 10, NaHCO_3_ 26, NaH_2_PO_4_ 1.2, D-glucose 25) or NMDG cutting buffer (in mM: NMDG/HCl 135, KCl 1, MgCl_2_ 1.5, Choline HCO_3_ 20, KH_2_PO_4_ 1.2, D-glucose 10, CaCl_2_ 0.5) [8]. Slices were recovered at 35°C for 20 min in standard artificial cerebrospinal fluid (ACSF) (in mM: NaCl 119, NaHCO_3_ 26, D-glucose 20, KCl 2.5, NaH_2_PO_4_ 1, MgSO_4_ 1.3, CaCl_2_ 2.5, saturated with carbogen, 95% O_2_, and 5% CO_2_) and then stored in carbogenated ACSF at room temperature until further use (1–7 h).

### Electrophysiology

Standard whole-cell voltage-clamp recordings were carried out at 30 ± 2°C in a recording chamber continuously (2 mL/min) perfused with ACSF. L2/3 or CA1 pyramidal neurons were visually identified with infrared-differential interference contrast microscopy. Glass pipettes (3–5 MΩ) were filled with Cs-based internal solution (in mM: CsMeSO_3_ 133, HEPES 10, TEA-OH 10, EGTA 0.25, D-glucose 10, MgCl_2_ 2, QX314-Cl 5, Na-ATP 4, Na-GTP 0.3, pH 7) or (in mM: Cs-gluconate 120, HEPES 20, EGTA 0.4, NaCl 2.8, TEA-Cl 5, Mg-ATP 4, Na-GTP 0.3, pH 7.2). The input and series resistance were monitored throughout the recording by applying a short hyperpolarizing voltage step before synaptic stimulation. Only cells with a series resistance smaller than 30 MΩ and changes of series and input resistance of less than 20% were used for analysis. Axons were stimulated in L4 with theta-glass bipolar electrodes filled with ACSF. Data were filtered at 3 kHz and collected with custom routines in Igor (Wavemetrics), using an ELC-03XS amplifier (NPI) and digitized at 10 kHz with an ITC-18 (HEKA). Glutamatergic transmission was isolated pharmacologically with 50 μM picrotoxin-supplemented ACSF and polysynaptic activity prevented in AMPA/NMDA ratio recordings with 1 μM 2-Chloroadenosine. Ten micromolar 2,3-dihydroxy-6-nitro-7-sulfamoyl-benzo[f]quinoxaline (NBQX) was supplemented to block AMPA receptors or 1 μM tetrodotoxin (TTX) to record mEPSCs [19]. For mEPSCs, 400 events of each cell were recorded, sorted by mEPSC amplitude size or inter-event interval time, and binned in 20 bins. Averages of bin values from different cells were plotted as cumulative probability plots.

### Synaptic failure analysis

The stimulation strength was adjusted such that successes and failures of AMPA receptor responses at a holding potential (V_h_) = −60 mV could be clearly visually identified. At a V_h_ = +40 mV, we then measured a composite response mediated by AMPA and NMDA receptors. Percent silent synapses were calculated using the equation 1 − ln(*F*_−60_)/ln(F_+40_), in which *F*_–60_ was the failure rate at −60 mV and *F*_+40_ was the failure rate at +40 mV [35,90]. As the failure rate is additionally dependent on the release probability, the stochastic nature of synaptic vesicle release will create variability in the failure rates at either V_h_, which may lead to mathematically negative fractions of silent synapses in individual trials. Whether synapses lacking NMDA receptors also contribute to the failure rate, as they exist, e.g., in Purkinje cells, is not known [119].

### AAV transduction

To generate sh93, we cloned the shRNA expression cassette from the lentiviral vector sh93 ms into the AAV vector AAV-sh95 to obtain pA_tn93b_CAGW [8,19]. An AAV with an shRNA against luciferase or with an expression cassette for EGFP only was used as a control for shRNA expression or AAV transduction, respectively [8]. To generate the AAV overexpressing PSD-95α, GFP-tagged PSD-95 was cloned from a lentiviral vector into the AAV vector under the CAG promoter to obtain pA_EB_CAGW_p95GFP [19]. To generate the AAV overexpressing PSD-93α2, the cDNA was cloned from a lentiviral vector into a dual promotor AAV vector, a gift from Dr. Yingxi Lin (Addgene 84474) [19,120]. The promoters were replaced by the mouse RNA polymerase II promoter to drive red fluorescent protein (RFP) expression and mouse CaMKIIα promoter to drive PSD-93 expression, named pA_R3A_Kp93a2 or pA_R3Ash95_Kp93a2, with, additionally, an miR30-based shRNA targeting PSD-95 in the 3′ UTR of the RFP. AAVs were produced based on described procedures, pseudo-typed with the capsid for AAV8, and purified by iodixanol gradient centrifugation [8]. A total of 80 nL of concentrated AAV was injected bilaterally into V1 of P0 mice, with surgical procedures as described previously [8]. The stereotactic coordinates for V1 were from lambda for P0: ±1.5 mm, +0.1 mm, and −0.8 mm.

### Subcellular fractionation

Subcellular fractions of a crude synaptosomal pellet were prepared as described previously [42]. Ten 1-mm-diameter punches of the visual cortex from brain slices were homogenized in 10 volumes of homogenization buffer (4 mM HEPES/NaOH pH 7.4, 320 mM sucrose), and after differential centrifugation, the crude synaptosomal pellet (P2) was resuspended with 1% SDS and adjusted to 1 μg/μL in SDS sample buffer. The protein extracts for the developmental profile were obtained from a previous study [8].

### Antibodies and quantitative western blotting

A total of 10–30 μg of protein was separated on Bis-Tris polyacrylamide gels and transferred on nitrocellulose membranes [42]. Protein bands were decorated with the following primary antibodies: PSD-93 (N18/30), PSD-95 (K28/43), SAP97 (K64/15), SAP102 (N19/2), Kv1.1 (K20/78), mortalin (N52A/42), GluA2 (L21/32), GluN2B (N59/36), vGluT1 (N28/9), GKAP (N238/31), CASK (K56A/50; all mouse from UC Davis/NIH NeuroMab), endocannabinoid receptor 1 (#258003), Munc13-1 (#126104; rabbit and guinea pig from Synaptic Systems), GluN2A (#05–901), GluA1 (#ABN241), GluN3A (#07–356), phospho-S295 PSD-95 (#04–1066; rabbit from Millipore), GluA3 (EP813Y), GluA4 (EPR2512[2]), phospho-S845 GluA1 (EPR2148; rabbit from Abcam), α-Synuclein, Rab3A, Rab3B, Synaptobrevin 2, Syntaxin 1a, Synaptotagmin 1, Synapsin 1, and Synaptopyhsin as described previously [121]. Bands were detected by the secondary antibodies goat anti-mouse Alexa 680 (Invitrogen); goat anti-rabbit Alexa 680 (Invitrogen); goat anti-mouse IR800 (Li-COR Biosciences); and goat anti-rabbit IR800 (Li-COR Biosciences), visualized, and quantified with an infrared fluorescence scanner. Band intensities for each sample and protein were normalized to the average of the control condition on the same blot to obtain a relative amount for each sample, which could be compared across different blots. Using previously described samples [8], for the developmental profile, band intensities for each sample and protein were normalized to the intensity at P90.

### Immunhistochemistry of 0.5-μm thin resin sections

One-millimeter-diameter punches from visual cortex of mouse brain slices (about 500 μm thick) were flash frozen in liquid nitrogen–cooled isopentane [50]. Lyophilization of the sample was typically performed in a vacuum of about 10^−5^ Pa for about 24 h. Samples were infiltrated with EPON resin at 22°C with degassing for 24 h. The resin-embedded samples were cured at 60°C for 24 h. After trimming the tissue block, 0.5-μm thin sections were cut with a diamond knife and collected on a glass slide. The EPON resin was removed by successive incubations with 30% Na-methanolate (in methanol) for 10 min, 50% xylol (in methanol) for 10 min, twice in acetone for 10 min, H_2_O for 10 min, and PBS for 10 min. Primary and secondary antibody incubations were performed as described [61]. Labeled samples were imaged on a Zeiss LSM 800 confocal microscope with a 63× objective. We acquired 7–10 mages (10.13 μm × 10.13 μm) for each semi-thin slice and analyzed 3–5 slices per animal. Images were analyzed with Fiji, and puncta-like objects were identified automatically by intensity and size [122]. The threshold of individual object size was set at 200 pixel^2^ (about 7,200 nm^2^, pixel size: 6 nm). Images were then processed manually, and fused puncta were segmented manually. Synapses were defined as puncta with overlapping (>5%) fluorescence signals of Munc13-1 and PSD-93 or PSD-95.

### Looming test

We used a custom-built version of the looming test [58] to check the defensive response of PSD-93/95 dKO mice to looming visual stimuli. The setup consisted of an acrylic box (48 cm × 48 cm × 30 cm) with a hut (20 cm × 12 cm) in a corner. Bottom, hut, and three walls were opaque; the front wall was translucent. The ceiling LCD monitor (HP Z22i IPS) displayed the looming stimuli (expanding black circles) as soon as a mouse entered a field in the center of the arena. The visual stimulus imitates an approaching predator: a black disc of 2-degree diameter on white background expands to 20 degrees within 250 ms and persists for another 250 ms, followed by a 500-ms break. Once triggered, the stimulus loops 15 times. Infrared LEDs in the ceiling illuminate the interior of the arena, invisible to the human and rodent eye but recordable, with a camera (Logitech, c920 HD Pro Webcam; with removed infrared filter) positioned outside of the setup, filming through the translucent wall. A mouse was placed in the arena and given 10 min to adapt to the new environment. In the subsequent test phase, we recorded whether an animal reacted to the stimulus or not. Each animal was tested maximally three times, only once per day, with at least 1 day of break in between tests.

### Running wheel

To check basic activity of PSD-93/95 dKO mice, we transferred animals for 24 h to a standard cage (22 cm × 37 cm × 15 cm) equipped with a running wheel [123], and calculated individual running distances. Each mouse was tested three times, with at least 24 h recovery between two measures.

### Cylinder test

To quantify exploratory behavior and activity, we adapted a cylinder test from the Behavioral and Functional Neuroscience Laboratory of the Stanford School of Medicine (http://med.stanford.edu/sbfnl/services/bm/sm/CylinderTest.html). Briefly, a mouse was placed in a glass cylinder (diameter = 14 cm, height = 21 cm). The forelimb contacts against the wall within 180 s were counted. To avoid conditioning, the test was only performed once per animal.

### Visual acuity

Visual acuity of the mice was assessed using the VWT, a visual discrimination task that is based on reinforcement learning [57,124]. For this task, mice were initially trained to distinguish a low spatial frequency vertical sine wave grating (0.086 cycles/degree) from equiluminant gray, and then their ability to recognize successively higher spatial frequencies was tested. The apparatus consists of a trapezoidal-shaped water-filled pool with two monitors placed side by side at one end. An escape platform that was invisible to the mice was placed below the monitor, on which the rewarded stimulus (grating) was projected. The position of the grating and the platform were alternated in a pseudorandom sequence over the training and test trials. Once 90% accuracy was achieved, the discrimination threshold was determined by increasing the spatial frequency of the grating until performance fell below 70% accuracy. The highest spatial frequency at which 70% accuracy was achieved was taken as the visual acuity threshold.

### Orientation discrimination

To measure orientation discrimination in mutant mice and WT controls, we used a variation of the published VWT [57,124]. Initially, mice were trained to distinguish vertical from horizontal gratings of a low spatial frequency (0.086 cycles/degree), and then their ability to recognize increasingly smaller orientation differences was tested by decreasing the difference in orientation of the two gratings in 5° steps until accuracy fell below 70% accuracy. The smallest orientation difference at which 70% accuracy was achieved was taken as the orientation discrimination threshold.

### Learning behavior

Only mice that learned the respective task (visual acuity or orientation discrimination) first were included in the “blocks to learn” analyses, i.e., our quantification of learning behavior only included naïve mice with respect to VWT training. This was done because learning one of the tasks often resulted in an accelerated learning of a second task [125]. Therefore, numbers of mice for this particular quantification of learning behavior were smaller compared with the two vision tests.

### MD

The right eye (contralateral to the recorded hemisphere) was deprived of vision for four d according to published protocols [30,126]. Briefly, mice were anesthetized with 2% isoflurane in 1:1 O_2_/N_2_O. Lid margins were trimmed and an antibiotic gel (gentamicin) was applied. The eye was closed with two mattress sutures. Mice were checked daily to make sure that the eyes remained closed. After MD, the mice were returned to their home cages.

### Optical imaging of intrinsic signals and visual stimuli

After initial anesthesia with 2% halothane in a mixture of 1:1 O_2_/N_2_O, the mice received atropine (Franz Köhler, 0.1 mg/mouse, s.c.), dexamethasone (Ratiopharm, 0.2 mg/mouse, s.c.) and chlorprothixene (Sigma, 0.2 mg/mouse, i.m.). In addition, lidocaine (2% xylocain jelly) was applied locally to all incisions. The mice were placed in a stereotaxic frame, their body temperature was maintained at 37°C, and electrocardiograph leads were attached to monitor the heart rate throughout the experiment. Anesthesia was maintained with 0.6%–0.8% halothane in a mixture of 1:1 O_2_/N_2_O applied through a tube over the nose. We incised the skin to expose the visual cortex of the left hemisphere, and low-melting-point agarose (2.5% in 0.9% NaCl) and a glass coverslip were placed over the exposed area. To avoid dehydration of the mouse during the experiment, we injected 0.2 mL saline (0.9%, s.c.).

Mouse visual cortical responses were recorded through the skull using the Fourier imaging method developed by Kalatsky and Stryker and optimized for the assessment of OD plasticity by Cang and colleagues [126,127]. The experimenter was blinded for the genotype of the recorded mouse. Briefly, a temporally periodic stimulus was continuously presented to the animal and the cortical responses at the stimulus frequency was extracted by Fourier analysis. Optical images of intrinsic cortical signals were obtained using a Dalsa 1M30 CCD camera (Dalsa, Waterloo, Canada) controlled by custom software. Using a 135 mm × 50 mm tandem lens configuration (Nikon, Melville, NY), we imaged a cortical area of 6.4 × 6.4 mm^2^. The surface vascular pattern and intrinsic signal images were visualized with illumination wavelengths set by a green (550 ± 10 nm) or red (610 ± 10 nm) interference filter, respectively. After acquisition of a surface image, the camera was focused 600 μm below the cortical surface. An additional red filter was interposed between the brain and the CCD camera. Frames were acquired at a rate of 30 Hz, temporally binned to 7.5 Hz, and stored as 512 × 512 pixel images after spatial binning of the camera image. Visual stimuli were displayed on an LCD monitor (Benq BL240 [LED], 1,920 × 1,080 @ 60 Hz), positioned 25 cm from the eyes, with the screen center aligned to the animal’s midline. Visual stimuli consisted of drifting horizontal or horizontal bars (2° wide). For imaging ODP, stimuli were restricted to the binocular visual field of the left V1 (−5° to +15° azimuth), and mice were stimulated through either the left or the right eye in alternation. For visualizing elevation and azimuth maps, we used full-field stimuli extending 94° horizontally and 62° vertically (see Fig 4) and contralateral eye stimulation.

### Data analysis

Visual cortical maps were calculated from the acquired frames by Fourier analysis to extract the signal at the stimulation frequency using custom software [127]. While the phase component of the signal was used for the calculation of retinotopy, the amplitude component represented the intensity of neuronal activation (expressed as fractional change in reflectance ×10^−4^) and was used to calculate OD (for details, see [126,128]). To quantify OD plasticity, an OD score of each pixel in the binocularly activated region was calculated as (C−I)/(C+I), with C and I representing the raw response magnitudes of each pixel to visual stimulation of the contralateral and ipsi, respectively. We then computed an ODI as the average of the OD scores of all responsive pixels. Consequently, ODI ranged from −1 to 1, with negative values representing ipsi and positive values representing contralateral dominance. We calculated ODIs from blocks of four runs; typically, we obtained at least five ODIs per animal. Experiments with less than three ODIs were discarded from further analyses. All ODIs of one animal were averaged for further quantification and data display. The ODIs were color-coded in a two-dimensional map of the OD scores (OD-map): cold colors represent negative values (ipsi dominance) and warm colors represent positive values (contralateral eye dominance).

### Statistical analyses

One- or two-factor ANOVA test with Tukey post hoc test was used for comparisons between genotype and developmental time point or groups of more than three, respectively. Two group comparisons were performed with two-tailed *t* test or MW test for data without normal distribution. Distributions of mEPSC data were compared with the KS test. All intra- and inter-group comparisons of the optical imaging data were done by a two-tailed *t* test or one-factor ANOVA test with Bonferroni post hoc test. Learning in the VWT was analyzed with Mantel-Cox survival analyses by comparing genotypes on number of blocks required to reach ≥90% accuracy. The levels of significance were set as *p < 0.05, **p < 0.01, ***p < 0.001. Data are represented as means ± SEM.

## Supporting information

S1 Fig Summary graph of time course of silent synapse maturation. The fraction of silent synapses in V1 of layer 4 onto layer 2/3 pyramidal neurons projections is plotted against the age of mice. Symbols are staggered to facilitate visibility. Data are from Figs 1 and 2. V1, primary visual cortex.
(TIF)

S2 Fig V1 activity and retinotopic maps in PSD-93 KO mice are not significantly altered. (A, D) Schematic presentation of an intrinsic optical imaging experiment, with visual stimulation of the contralateral eye of the mouse with moving horizontal (A) or vertical bars (D). (B, C, E, F) Optically recorded activity and retinotopic maps in the left V1 of WT and PSD-93 KO mice. Both elevation (B and C) and azimuth maps (E and F) resulting from visual stimulation of the contralateral eye of the mouse with moving horizontal (A) or vertical bars (D) are illustrated. Grayscale coded response magnitude maps (upper) and color-coded phase maps (lower) are shown. The magnitude of the optical responses (V1 activation) is expressed as fractional change in reflection ×10^−4^; the grayscale bar in B applies to all amplitude maps. Retinotopic maps are color-coded according to the schemes in A and D (scale bar for all panels with maps, 1 mm). (G–J) Quantification of V1 activation (G and I) and map quality/scatter (H and J) for WT and PSD-93 KO mice. Number of mice is indicated in the foot of the bar. Underlying data for this figure can be found in S1 Data. KO, knock-out; PSD, postsynaptic density; V1, primary visual cortex; WT, wild-type.
(TIF)

S3 Fig V1 activity and retinotopic maps with AAV-sh93 expression are not significantly altered. Layout and data display are as in S2 Fig. (A, D) V1 was activated by stimulating the contralateral eye with moving horizontal (A) or vertical bars (D). (B, C, E, F) Optically recorded activity and retinotopic maps in the left V1 of WT mice with shLC (B, E) or with sh93 (C, F). Scale bar for all panels with maps, 1 mm. (G–J) Quantification of V1 activation (G and I) and map quality/scatter (H and J) for WT mice with AAV-shLC (green) or AAV-sh93 (red). AAV, adeno-associated viral vector; sh93, short hairpin RNA against PSD-93; shLC, short hairpin RNA against luciferase; V1, primary visual cortex; WT, wild-type.
(TIF)

S4 Fig Synaptic parameters of PSD-93 and PSD-95 KO mice. (A) Release probability of the layer 4 to layer 2/3 pyramidal cell pathway in V1 was assessed with the open channel NMDA receptor blocker MK801 in P28 WT (black) and PSD-93 KO mice (red). Pyramidal neurons were voltage clamped at V_h_ = +40 mV and NMDA receptor EPSCs pharmacologically isolated (inset). The progressive reduction in the peak amplitude is plotted against the sweep number. Three mice, each with *n*_WT_ = 10, *n*_93KO_ = 11 neurons, per group. Scale bar: 50 pA, 50 ms. Underlying data for this figure can be found in S1 Data. (B, C) Asynchronous release was forced by substituting Ca^2+^ for Sr^2+^ in the ACSF to measure quantal responses in the layer 4 to layer 2/3 pyramidal cell pathway in V1. Sample trace (B) and cumulative probability distribution of evoked quantal EPSC amplitude are illustrated. Data were binned as described for mEPSC analysis. Average quantal EPSC amplitude is illustrated in inset. Number of cells in the foot of the bar. Scale bar: 25 pA, 100 ms. Underlying data for this figure can be found in S1 Data. (D, E) mEPSC recordings with cumulative probability graph of mEPSC amplitude (D) and IEI (E) for WT (gray) and PSD-95 KO (blue) mice at P23 (P20–P26). Average mEPSC amplitude (D) and IEI (E) are illustrated in the inset. Number of layer 2/3 pyramidal neurons is indicated in the foot of the bar. KS test for equal distribution or *t* test for difference of means, **p < 0.01. Data for WT are the same as Fig 2. Underlying data for this figure can be found in S1 Data. (F, G) AMPA receptor EPSC and NMDA receptor EPSC ratio, measured as peak current at V_h_ = −60 mV and 50 ms after peak current at V_h_ = +40 mV, respectively. Sample traces (F) and summary graph (G) are illustrated. Arrow depicts time point of NMDAR EPSC measurement, when AMPAR EPSC is returned to baseline values. Number of cells is indicated in the foot of the bar. Scale bar: 100 pA, 100 ms. **p < 0.01. Underlying data for this figure can be found in S1 Data. ACSF, artificial cerebrospinal fluid; AMPA, α-amino-3-hydroxy-5-methyl-4-isoxazole propionic acid; AMPAR, AMPA-type glutamate receptor; EPSC, excitatory postsynaptic current; IEI, inter-event interval; KO, knock-out; KS, Kolmogorov-Smirnov; m, miniature; NMDA, N-methyl-D-aspartate; NMDAR, NMDA-type glutamate receptor; P, postnatal day; PSD, postsynaptic density; V_h_, holding potential; V1, primary visual cortex; WT, wild-type.
(TIF)

S5 Fig Synapse density in V1 of PSD-93 and PSD-95 KO mice. (A-C) Immunofluorescence labeling of semi-thin sections with overview (top two rows) and enlargement of boxed area (bottom two rows) of mouse (P40) visual cortex for PSD-95 (green), PSD-93 (blue), and Munc13-1 (red) in WT (A), PSD-95 KO (B), and PSD-93 KO mice (C). Upper panels illustrate fluorescence for single channels and lower panels for two channels with PSD-95/93 (left) PSD-93/Munc13-1 (middle) and PSD-95/Munc13-1 (right). Arrowheads depict puncta in PSD-93 KO, which are decorated with PSD-93 antibody and colocalize with Munc13-1 positive puncta. For quantification of synapse density, see Fig 6. Scale bar: 2 μm. (D) Western blot of crude synaptosomal fractions of cortex from WT, PSD-93, and PSD-95 KO mice. KO, knock-out; Munc13-1, Mammalian uncoordinated 13–1; P, postnatal day; PSD, postsynaptic density; V1, primary visual cortex; WT, wild-type.
(TIF)

S6 Fig Developmental profile of synaptic proteins in mouse V1 and changes of synaptic proteins in V1 of DR PSD-93 KO mice. (A-G) Developmental profile of synaptic proteins GKAP (A, B, blue), phospho-S295 PSD-95 (A, B, turquoise), GluN3A (A, B, gray), phospho-S845 GluA1 (A, B, green), Rab3B (C, D, blue), Synaptophysin (C, D, green), Rab3A (C, D, red), vGluT1 (C, D, gray), Synapsin 1 (E, F, gray), Synaptotagmin 1 (E, F, green), Syntaxin 1a (E, F, red), Synaptobrevin 2 (E, F, blue), CASK (G, H, green), endocanabinoid receptor 1 (G, H, blue), α-Synuclein (G, H, red), and Kv1.1 (G, H, gray) from crude synaptosomal fractions from V1 of WT mice. Sample blots (A, C, E, G) are illustrated for the indicated proteins, and quantified protein levels, normalized to the adult levels at P90, are plotted against the postnatal day (P, B, D, F, G). *n* = 4–5 (mice). (I) Comparison of synaptic protein levels from crude synaptosomal fractions from DR PSD-93 KO mice and NR WT mice at P28. Sample western blots illustrated on right. Protein levels were assessed as described in panel Fig 6F. Number of mice is indicated in the foot of the bar. *t* test, *p < 0.05. Underlying data for this figure can be found in S1 Data. CASK, Calcium/Calmodulin Dependent Serine Protein Kinase; CP, critical period; DR, dark-reared; EO, eye opening; GKAP, guanylate kinase-associated protein; Glu, glutamate receptor subunit; KO, knock-out; Kv, voltage-gated potassium channel; NR, normal-reared; P, postnatal day; PSD, postsynaptic density; Rab, ras-related protein; vGluT1, vesicular glutamate transporter 1; V1, primary visual cortex; WT, wild-type.
(TIF)

S7 Fig Hedonic motivation in PSD-93/95 double KO mice is impaired. (A) Voluntary running of WT (black) and PSD-93/95 double KO mice (orange) in running wheel (scheme on top). Average distance per 24 h for 3 consecutive days for each mouse is presented as a dot and the average as a horizontal line. *t* test, **p < 0.01. Underlying data for this figure can be found in S1 Data. (B) Cylinder test (scheme on top) to assess exploratory behavior by counting front paw contacts with wall after raising on hind paws during 180s. *t* test, **p < 0.01. Underlying data for this figure can be found in S1 Data. KO, knock-out; PSD, postsynaptic density; WT, wild-type.
(TIF)

S8 Fig V1 activity and retinotopic maps in PSD-93/95 dKO mice are not significantly altered. Layout and data display as in S2 Fig. (A, D) V1 was activated by stimulating the contralateral eye with moving horizontal (A) or vertical bars (D). (B, C, E, F) Optically recorded activity and retinotopic maps in the binocular segment of the left V1 of WT mice (B, E) or dKO mice (C, F). Scale bar for all panels with maps, 1 mm. (G–J) Quantification of V1 activation (G and I) and map quality/scatter (H and J) for WT mice (black) or dKO mice (orange). Underlying data for this figure can be found in S1 Data. dKO, double KO; PSD, postsynaptic density; V1, primary visual cortex; WT, wild-type.
(TIF)

S1 Data Underlying data for Figs 1–8 and S1–S8. (XLSX)

## Abbreviations

AAV: adeno-associated viral vector
AMPA: α-amino-3-hydroxy-5-methyl-4-isoxazole propionic acid
AMPAR: AMPA-type glutamate receptor
ASD: autism spectrum disorder
BDNF: brain-derived neurotrophic factor
CA1: Cornu Ammonis 1
contra: contralateral eye
dKO: double KO
DLG: disc-large
DR: dark-reared
EPSC: excitatory postsynaptic current
GABA: γ-aminobutyric acid
GFP: green fluorescent protein
Glu: glutamate receptor subunit
ipsi: ipsilateral eye
jODP: juvenile ODP
KD: knock-down
KO: knock-out
KS: Kolmogorov-Smirnov
Kv: voltage-gated potassium channel subunit
L4: layer 4
LTP: long-term synaptic potentiation
m: miniature
MAGUK: membrane-associated guanylate kinase
MD: monocular deprivation
mPFC: medial prefrontal cortex
Munc13-1: Mammalian uncoordinated 13–1
MW: Mann-Whitney
NBQX: 2,3-dihydroxy-6-nitro-7-sulfamoyl-benzo[f]quinoxaline
NDMA: N-methyl-D-aspartate
NMDAR: NMDA-type glutamate receptor
NR: normal-reared
OD: ocular dominance
ODI: OD index
ODP: OD plasticity
P: postnatal day
PrL: prelimbic cortex
PSD: postsynaptic density
Rab: Ras-related protein
SAP: synapse-associated protein
SAPAP: SAP90/PSD-95–associated protein
Shank: Src homology domain and multiple Ankyrin repeat domains protein
shLC: short hairpin RNA against luciferase
sh93: short hairpin RNA against PSD-93
sh95: short hairpin RNA against PSD-95
STEP: striatal enriched protein tyrosine phosphatase
TTX: tetrodotoxin
VWT: visual water task
V1: primary visual cortex
vGlut: vesicular glutamate transporter
V_h_: holding potential
WT: wild-type
