# Supplementary figures and images for: An opposing function of paralogs in balancing developmental synapse maturation
Source: PLoS Biol. 2018 Dec 26;16(12):e2006838. doi: 10.1371/journal.pbio.2006838 (PMC6324823; doi:10.1371/journal.pbio.2006838)

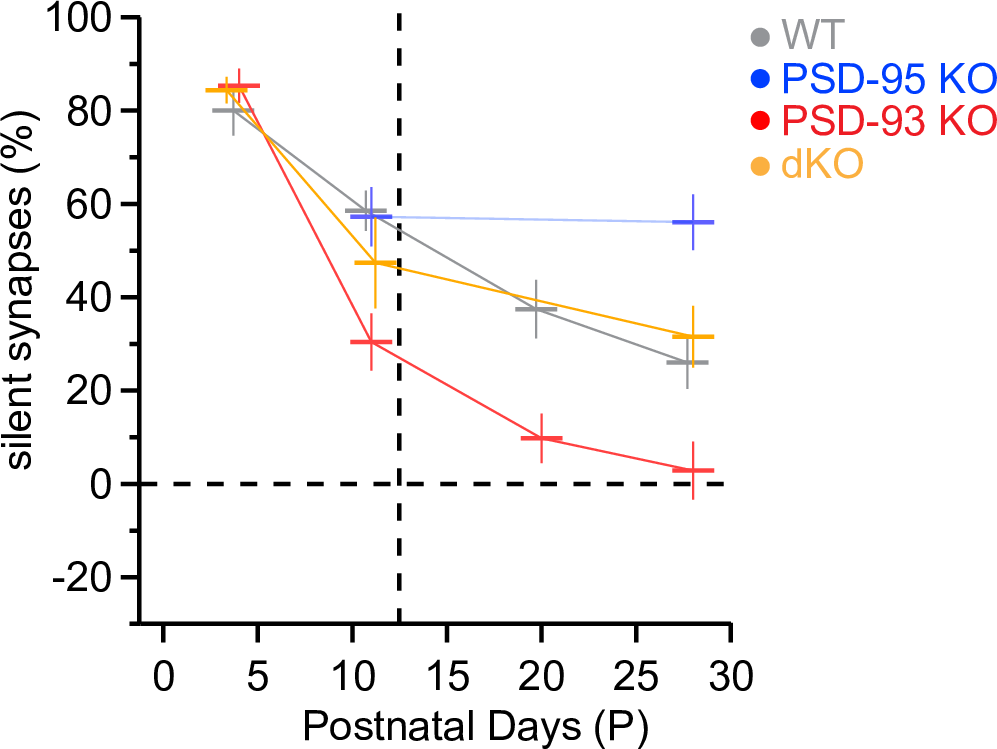

Supplement: S1 Fig — The fraction of silent synapses in V1 of layer 4 onto layer 2/3 pyramidal neurons projections is plotted against the age of mice. Symbols are staggered to facilitate visibility. Data are from Figs 1 and 2. V1, primary visual cortex. (TIF) [file pbio.2006838.s001.tif]

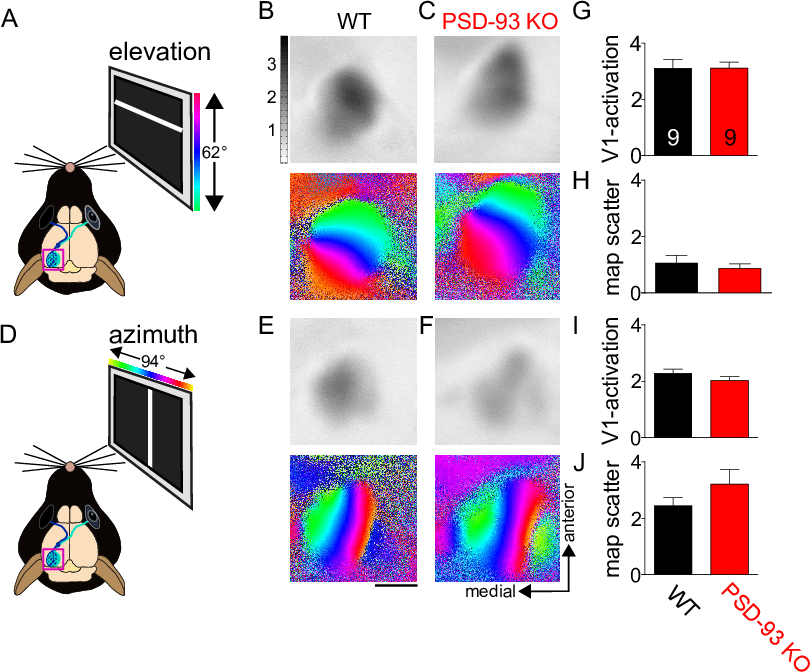

Supplement: S2 Fig — (A, D) Schematic presentation of an intrinsic optical imaging experiment, with visual stimulation of the contralateral eye of the mouse with moving horizontal (A) or vertical bars (D). (B, C, E, F) Optically recorded activity and retinotopic maps in the left V1 of WT and PSD-93 KO mice. Both elevation (B and C) and azimuth maps (E and F) resulting from visual stimulation of the contralateral eye of the mouse with moving horizontal (A) or vertical bars (D) are illustrated. Grayscale coded response magnitude maps (upper) and color-coded phase maps (lower) are shown. The magnitude of the optical responses (V1 activation) is expressed as fractional change in reflection ×10−4; the grayscale bar in B applies to all amplitude maps. Retinotopic maps are color-coded according to the schemes in A and D (scale bar for all panels with maps, 1 mm). (G–J) Quantification of V1 activation (G and I) and map quality/scatter (H and J) for WT and PSD-93 KO mice. Number of mice is indicated in the foot of the bar. Underlying data for this figure can be found in S1 Data. KO, knock-out; PSD, postsynaptic density; V1, primary visual cortex; WT, wild-type. (TIF) [file pbio.2006838.s002.tif]

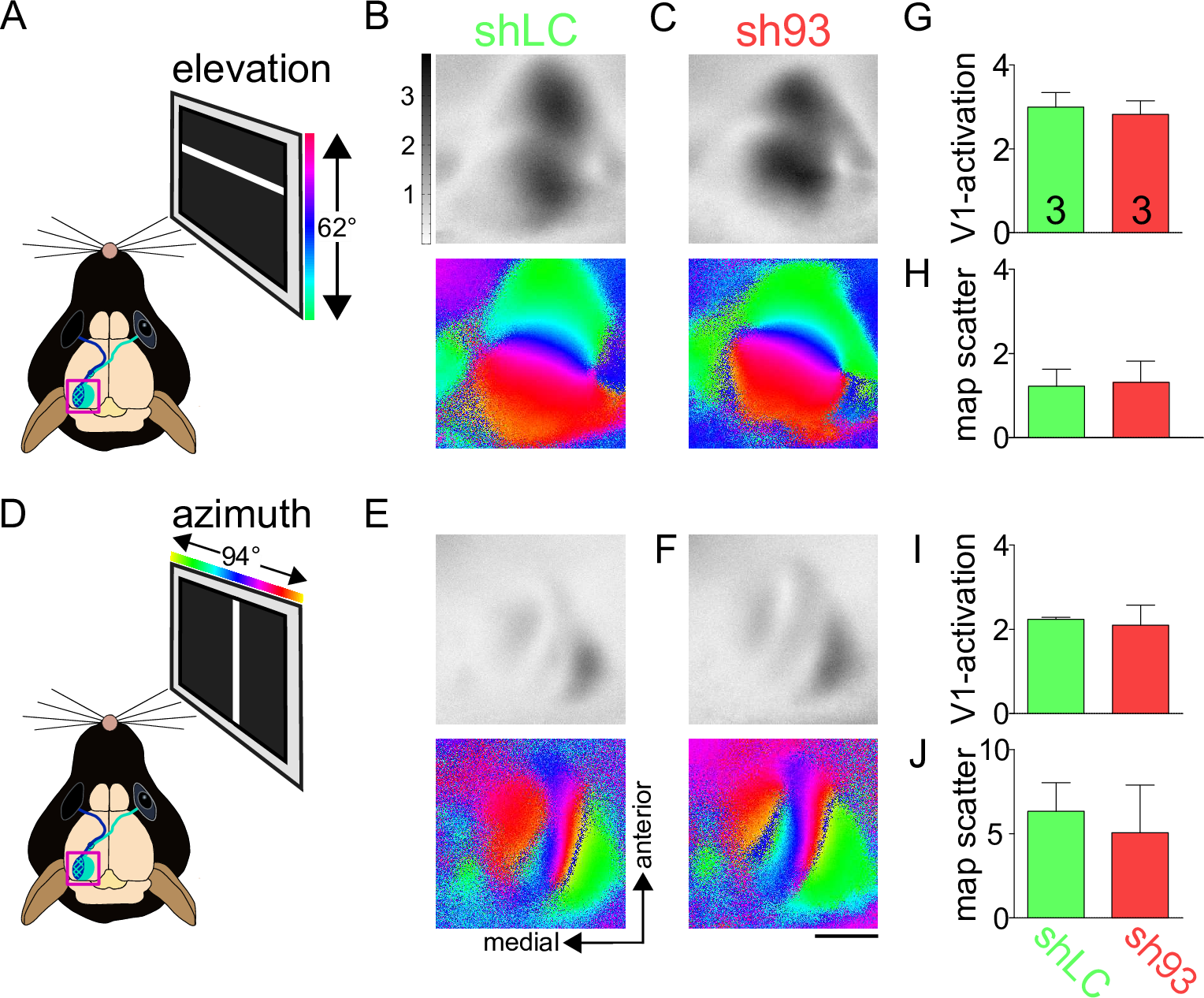

Supplement: S3 Fig — Layout and data display are as in S2 Fig. (A, D) V1 was activated by stimulating the contralateral eye with moving horizontal (A) or vertical bars (D). (B, C, E, F) Optically recorded activity and retinotopic maps in the left V1 of WT mice with shLC (B, E) or with sh93 (C, F). Scale bar for all panels with maps, 1 mm. (G–J) Quantification of V1 activation (G and I) and map quality/scatter (H and J) for WT mice with AAV-shLC (green) or AAV-sh93 (red). AAV, adeno-associated viral vector; sh93, short hairpin RNA against PSD-93; shLC, short hairpin RNA against luciferase; V1, primary visual cortex; WT, wild-type. (TIF) [file pbio.2006838.s003.tif]

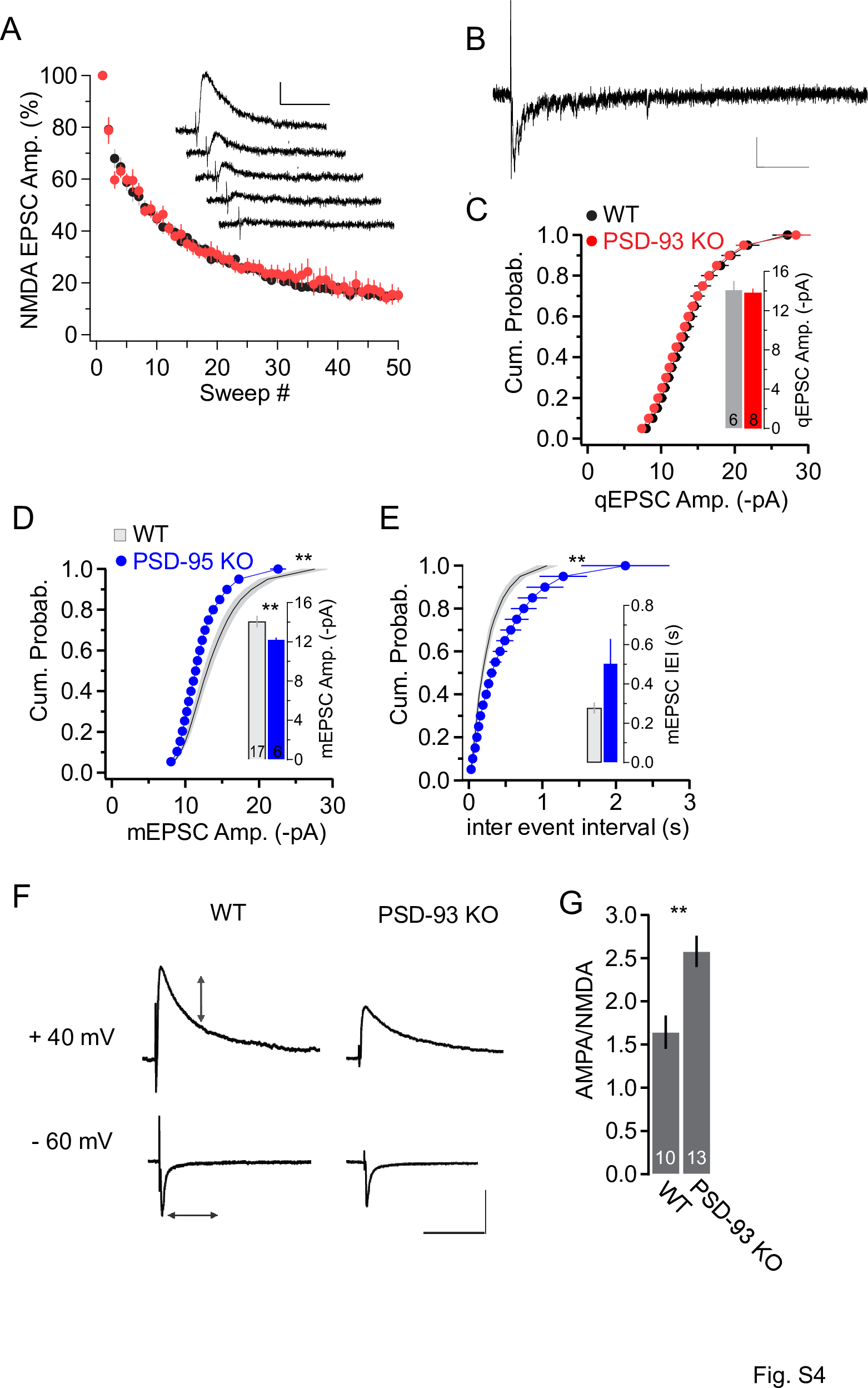

Supplement: S4 Fig — (A) Release probability of the layer 4 to layer 2/3 pyramidal cell pathway in V1 was assessed with the open channel NMDA receptor blocker MK801 in P28 WT (black) and PSD-93 KO mice (red). Pyramidal neurons were voltage clamped at Vh = +40 mV and NMDA receptor EPSCs pharmacologically isolated (inset). The progressive reduction in the peak amplitude is plotted against the sweep number. Three mice, each with nWT = 10, n93KO = 11 neurons, per group. Scale bar: 50 pA, 50 ms. Underlying data for this figure can be found in S1 Data. (B, C) Asynchronous release was forced by substituting Ca2+ for Sr2+ in the ACSF to measure quantal responses in the layer 4 to layer 2/3 pyramidal cell pathway in V1. Sample trace (B) and cumulative probability distribution of evoked quantal EPSC amplitude are illustrated. Data were binned as described for mEPSC analysis. Average quantal EPSC amplitude is illustrated in inset. Number of cells in the foot of the bar. Scale bar: 25 pA, 100 ms. Underlying data for this figure can be found in S1 Data. (D, E) mEPSC recordings with cumulative probability graph of mEPSC amplitude (D) and IEI (E) for WT (gray) and PSD-95 KO (blue) mice at P23 (P20–P26). Average mEPSC amplitude (D) and IEI (E) are illustrated in the inset. Number of layer 2/3 pyramidal neurons is indicated in the foot of the bar. KS test for equal distribution or t test for difference of means, **p < 0.01. Data for WT are the same as Fig 2. Underlying data for this figure can be found in S1 Data. (F, G) AMPA receptor EPSC and NMDA receptor EPSC ratio, measured as peak current at Vh = −60 mV and 50 ms after peak current at Vh = +40 mV, respectively. Sample traces (F) and summary graph (G) are illustrated. Arrow depicts time point of NMDAR EPSC measurement, when AMPAR EPSC is returned to baseline values. Number of cells is indicated in the foot of the bar. Scale bar: 100 pA, 100 ms. **p < 0.01. Underlying data for this figure can be found in S1 Data. ACSF, artificial cerebrospinal fluid; [file pbio.2006838.s004.tif]

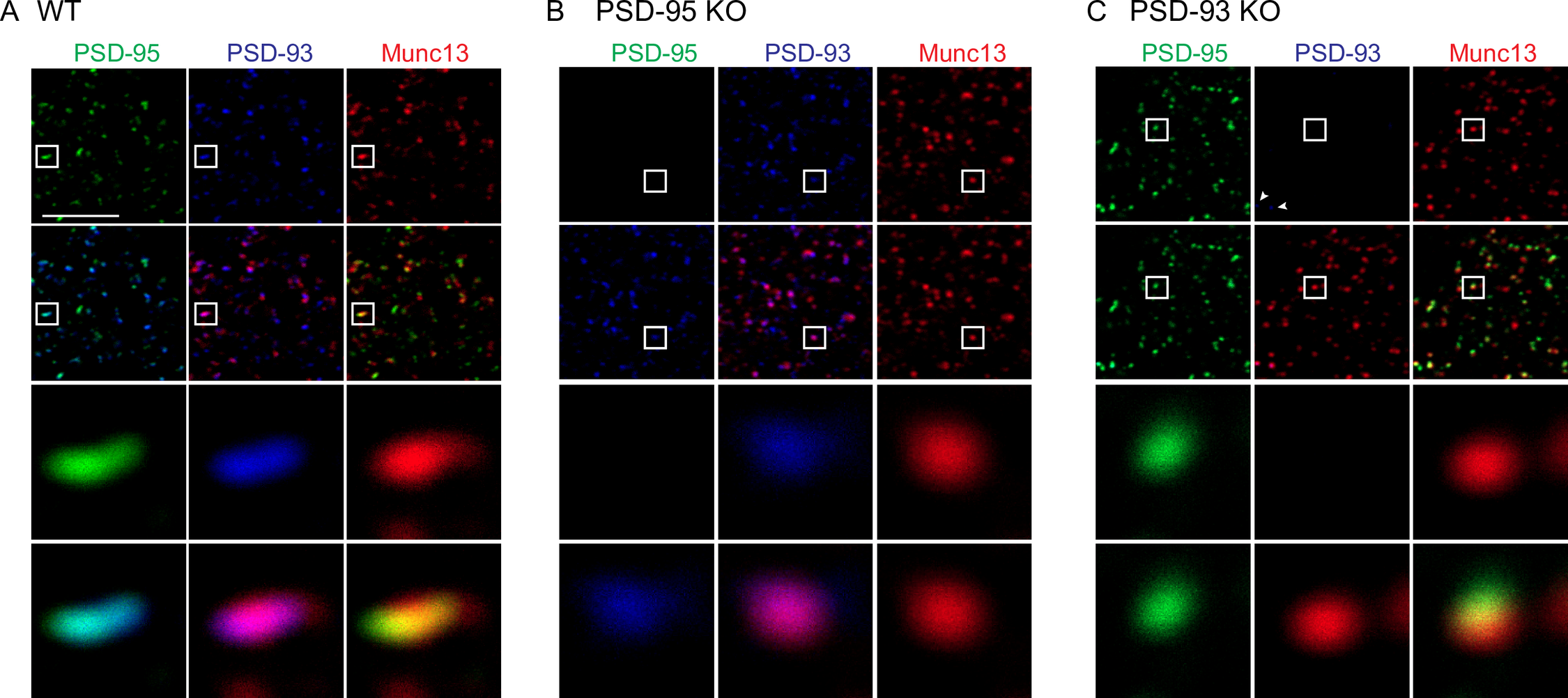

Supplement: S5 Fig — (A-C) Immunofluorescence labeling of semi-thin sections with overview (top two rows) and enlargement of boxed area (bottom two rows) of mouse (P40) visual cortex for PSD-95 (green), PSD-93 (blue), and Munc13-1 (red) in WT (A), PSD-95 KO (B), and PSD-93 KO mice (C). Upper panels illustrate fluorescence for single channels and lower panels for two channels with PSD-95/93 (left) PSD-93/Munc13-1 (middle) and PSD-95/Munc13-1 (right). Arrowheads depict puncta in PSD-93 KO, which are decorated with PSD-93 antibody and colocalize with Munc13-1 positive puncta. For quantification of synapse density, see Fig 6. Scale bar: 2 μm. (D) Western blot of crude synaptosomal fractions of cortex from WT, PSD-93, and PSD-95 KO mice. KO, knock-out; Munc13-1, Mammalian uncoordinated 13–1; P, postnatal day; PSD, postsynaptic density; V1, primary visual cortex; WT, wild-type. (TIF) [file pbio.2006838.s005.tif]

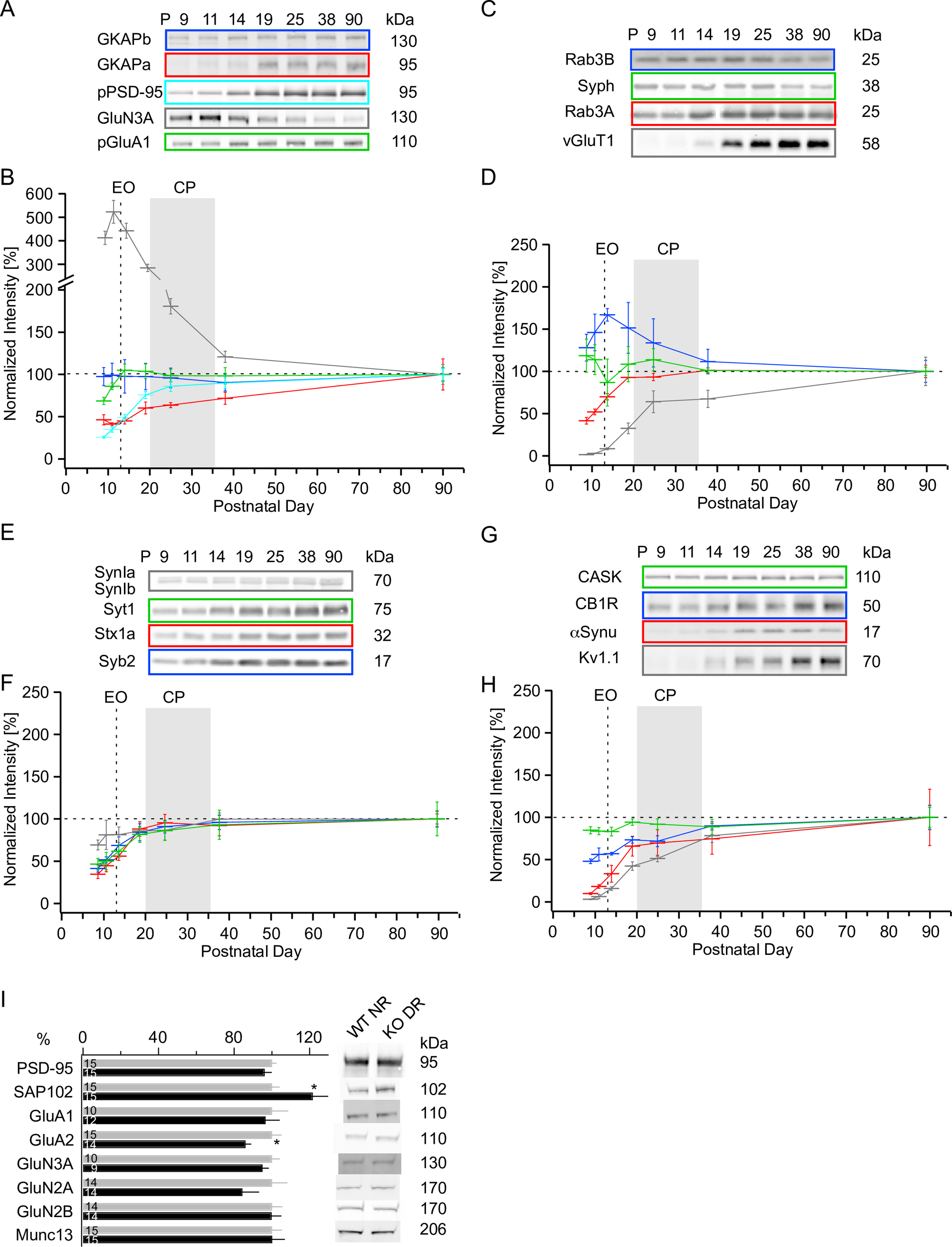

Supplement: S6 Fig — (A-G) Developmental profile of synaptic proteins GKAP (A, B, blue), phospho-S295 PSD-95 (A, B, turquoise), GluN3A (A, B, gray), phospho-S845 GluA1 (A, B, green), Rab3B (C, D, blue), Synaptophysin (C, D, green), Rab3A (C, D, red), vGluT1 (C, D, gray), Synapsin 1 (E, F, gray), Synaptotagmin 1 (E, F, green), Syntaxin 1a (E, F, red), Synaptobrevin 2 (E, F, blue), CASK (G, H, green), endocanabinoid receptor 1 (G, H, blue), α-Synuclein (G, H, red), and Kv1.1 (G, H, gray) from crude synaptosomal fractions from V1 of WT mice. Sample blots (A, C, E, G) are illustrated for the indicated proteins, and quantified protein levels, normalized to the adult levels at P90, are plotted against the postnatal day (P, B, D, F, G). n = 4–5 (mice). (I) Comparison of synaptic protein levels from crude synaptosomal fractions from DR PSD-93 KO mice and NR WT mice at P28. Sample western blots illustrated on right. Protein levels were assessed as described in panel Fig 6F. Number of mice is indicated in the foot of the bar. t test, *p < 0.05. Underlying data for this figure can be found in S1 Data. CASK, Calcium/Calmodulin Dependent Serine Protein Kinase; CP, critical period; DR, dark-reared; EO, eye opening; GKAP, guanylate kinase-associated protein; Glu, glutamate receptor subunit; KO, knock-out; Kv, voltage-gated potassium channel; NR, normal-reared; P, postnatal day; PSD, postsynaptic density; Rab, ras-related protein; vGluT1, vesicular glutamate transporter 1; V1, primary visual cortex; WT, wild-type. (TIF) [file pbio.2006838.s006.tif]

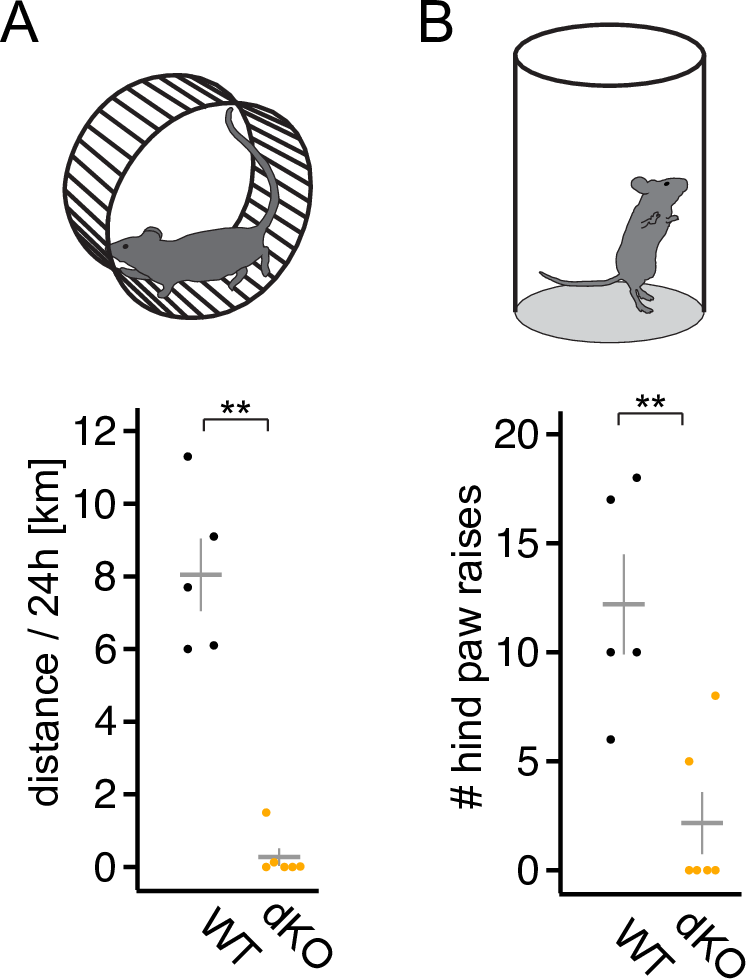

Supplement: S7 Fig — (A) Voluntary running of WT (black) and PSD-93/95 double KO mice (orange) in running wheel (scheme on top). Average distance per 24 h for 3 consecutive days for each mouse is presented as a dot and the average as a horizontal line. t test, **p < 0.01. Underlying data for this figure can be found in S1 Data. (B) Cylinder test (scheme on top) to assess exploratory behavior by counting front paw contacts with wall after raising on hind paws during 180s. t test, **p < 0.01. Underlying data for this figure can be found in S1 Data. KO, knock-out; PSD, postsynaptic density; WT, wild-type. (TIF) [file pbio.2006838.s007.tif]

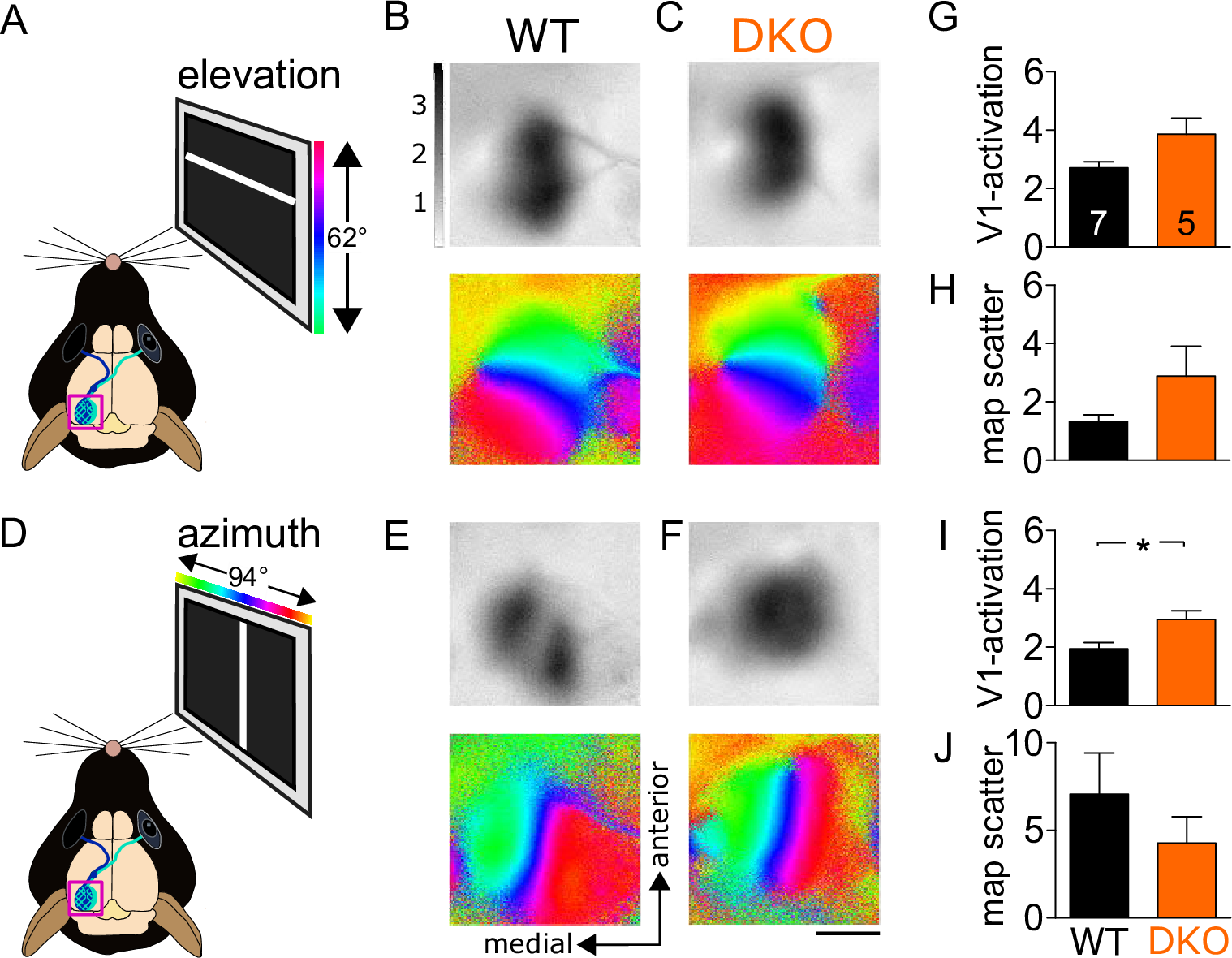

Supplement: S8 Fig — Layout and data display as in S2 Fig. (A, D) V1 was activated by stimulating the contralateral eye with moving horizontal (A) or vertical bars (D). (B, C, E, F) Optically recorded activity and retinotopic maps in the binocular segment of the left V1 of WT mice (B, E) or dKO mice (C, F). Scale bar for all panels with maps, 1 mm. (G–J) Quantification of V1 activation (G and I) and map quality/scatter (H and J) for WT mice (black) or dKO mice (orange). Underlying data for this figure can be found in S1 Data. dKO, double KO; PSD, postsynaptic density; V1, primary visual cortex; WT, wild-type. (TIF) [file pbio.2006838.s008.tif]
